# Computational Models of HIV-1 Resistance to Gene Therapy Elucidate Therapy Design Principles

**DOI:** 10.1371/journal.pcbi.1000883

**Published:** 2010-08-12

**Authors:** Sharon Aviran, Priya S. Shah, David V. Schaffer, Adam P. Arkin

**Affiliations:** 1 Department of Bioengineering, University of California Berkeley, Berkeley, California, United States of America; 2 Department of Chemical Engineering, University of California Berkeley, Berkeley, California, United States of America; 3 The Helen Wills Neuroscience Institute, University of California Berkeley, Berkeley, California, United States of America; 4 Physical Biosciences Division, Lawrence Berkeley Laboratory, Berkeley, California, United States of America; University of California San Diego, United States of America

## Abstract

Gene therapy is an emerging alternative to conventional anti-HIV-1 drugs, and can potentially control the virus while alleviating major limitations of current approaches. Yet, HIV-1's ability to rapidly acquire mutations and escape therapy presents a critical challenge to any novel treatment paradigm. Viral escape is thus a key consideration in the design of any gene-based technique. We develop a computational model of HIV's evolutionary dynamics in vivo in the presence of a genetic therapy to explore the impact of therapy parameters and strategies on the development of resistance. Our model is generic and captures the properties of a broad class of gene-based agents that inhibit early stages of the viral life cycle. We highlight the differences in viral resistance dynamics between gene and standard antiretroviral therapies, and identify key factors that impact long-term viral suppression. In particular, we underscore the importance of mutationally-induced viral fitness losses in cells that are not genetically modified, as these can severely constrain the replication of resistant virus. We also propose and investigate a novel treatment strategy that leverages upon gene therapy's unique capacity to deliver different genes to distinct cell populations, and we find that such a strategy can dramatically improve efficacy when used judiciously within a certain parametric regime. Finally, we revisit a previously-suggested idea of improving clinical outcomes by boosting the proliferation of the genetically-modified cells, but we find that such an approach has mixed effects on resistance dynamics. Our results provide insights into the short- and long-term effects of gene therapy and the role of its key properties in the evolution of resistance, which can serve as guidelines for the choice and optimization of effective therapeutic agents.

## Author Summary

A primary obstacle to the success of any anti-HIV treatment is HIV's ability to rapidly resist it by generating new viral strains whose vulnerability to the treatment is reduced. Gene therapies represent a novel class of treatments for HIV infection that may supplement or replace present therapies, as they alleviate some of their major shortcomings. The design of gene therapeutic agents that effectively reduce viral resistance can be aided by a quantitative elucidation of the processes by which resistance is acquired following therapy initiation. We developed a computational model that describes a patient's response to therapy and used it to quantify the influence of therapy parameters and strategies on the development of viral resistance. We find that gene therapy induces different clinical conditions and a much slower viral response than present therapies. These dictate different design principles such as a greater significance to the virus' competence in the absence of therapy. We also show that one can effectively delay emergence of resistance by delivering distinct therapeutic genes into separate cell populations. Our results highlight the differences between traditional and gene therapies and provide a basic understanding of how key controllable parameters and strategies affect resistance development.

## Introduction

With no HIV-1 vaccine or cure in sight, treating and controlling the virus continues to be a major global health concern [1], [2]. The advent of highly active antiretroviral therapy (HAART) has remarkably prolonged patients' survival, but has failed to eradicate the virus or to control the epidemic. In particular, HAART is a lifelong treatment, and as such presents major obstacles, including cumulative toxicities, severe side effects, a strict and complicated regimen, and problematic economics. Its major problem, however, is HIV-1's ability to escape it by developing drug-resistant mutants, which is further worsened by poor patient compliance [3]. Currently, the pace of development for new therapies lags behind HIV's rapid evolution of drug resistance, and alternative approaches are sought to either complement or replace HAART.

Gene therapy is an emerging and promising approach to treating HIV-1 infection, whereby engineered genes are delivered *ex vivo*, and potentially ultimately *in vivo*, into a patient's cells. They then act within these cells to disrupt the viral life cycle. Gene therapy offers the potential to attain sustained viral suppression and a restored immune system, with the added advantage of a simplified regimen, very few medical interventions, and reduced toxicities. To date, a plethora of potent gene-based inhibitors have been developed in the lab and some have undergone early-phase clinical trials (reviewed in [4]). While the trials demonstrated safety and feasibility, the infused gene-modified cells did not accumulate with time and consequently could not exert meaningful clinical effects [5], [6], [7]. Achieving therapeutic proportions of gene-modified cells *in vivo* is thus a necessary preliminary step for gene therapy's success. Ultimately, however, this approach must prove efficacious in the presence of viral resistance in order to qualify as a feasible therapeutic option. Indeed, as with HAART, viral escape is presently a major concern in the design of any gene-based technique [8], [9], [10], [11], and combinatorial gene cassettes are commonly developed as a means of limiting escape [12], [13], [14]. While the qualitative relations between key design parameters and viral escape are generally understood, a more rigorous quantitative investigation is essential to better understand the parameters' long-term effects under clinically-relevant conditions. The focus of this work is on a computational modeling approach to illustrate the contribution of therapy parameters and strategies to delaying the emergence of resistant virus in a patient.

Modeling HIV dynamics is by now a well-accepted tool for elucidating mechanisms of interest and for understanding viral evolution [15], [16], [17], [18]. A great deal of work has been published with regards to HAART, and has had much success largely due to its clinical validation against patient data. For novel treatments like gene therapy, however, substantial clinical data is not yet available. One must then resort to theoretical investigation as a much-needed step in therapy design. However, very few models have explored viral dynamics under gene therapy, and these have focused primarily on the response of virus that is sensitive or not resistant to the therapy [19], [20], [21]. Interestingly, this work revealed major deviations from HAART-like dynamics, thus underscoring a need for a dedicated model of viral resistance under gene therapy conditions.

Leonard *et al.* [22] developed a stochastic *in vitro* model that elucidates HIV's escape from RNA interference (RNAi) gene therapy. While powerful for studying escape *in vitro* [23], the model has several features that limit its relevance to *in vivo* scenarios. First, it focuses on RNAi therapies that degrade viral transcripts, an intervention that occurs after a cell has been infected and may thus not facilitate sufficient outgrowth of the gene-modified cells *in vivo*, as was later suggested in [21]. Conferring the modified cells with substantial outgrowth capacity is essential in any practical setting due to severe limitations on the fraction of cells that can be genetically modified [24], [25]. Other properties that diverge from *in vivo* conditions include simulations that often predict complete viral eradication [19], [21], and small population sizes that might under-represent minority viral strains [26], [27]. Since sustained viral replication and pre-existing mutants both play a crucial role in fueling resistance, they should be included in an *in vivo* model. Recently, von Laer *et al.*'s study [21] suggested that genes which inhibit early stages in the viral life cycle (by preventing cell binding, membrane fusion, reverse transcription, or integration) have the capacity to propel major cell expansion and therapeutic benefit. A variety of suitable gene-based techniques can be used, including RNAi- [28], [29], ribozyme- [13], zinc-finger nuclease- [30], and antibody-mediated [31] disruption of the CCR5 co-receptor, expression of fusion-inhibitory and binding-inhibitory peptides and of single-chain antibodies [32], and interference with capsid uncoating [33].

In this study, we developed a hybrid stochastic-deterministic approach for describing the evolution of HIV's resistance to early-stage gene-based inhibitors *in vivo*. We extended prior modeling work [19], [21] to incorporate a diverse viral population entailing varying degrees of sensitivity to therapy, and to account for the random effects that dominate early phases of resistance development. Our aim is to provide a general model that captures the commonalities of a broad range of technologies and that can be further adapted to faithfully describe any specific treatment. We apply the model to elucidate the general principles that govern resistance evolution and present extensive simulation results that quantify the tradeoffs between controllable therapy parameters. We show that the fundamentally different dynamics under gene therapy suggest different design guidelines from HAART's. Specifically, unlike HAART, in which drugs provide nearly-homogeneous protection to most cells, protected (gene-modified) and unprotected (untreated) cells co-exist under gene therapy. We find that this property can be harnessed to impede escape, provided that the mutations are associated with non-negligible fitness losses in non-modified cells.

We also investigate a novel delivery strategy to combat resistance, whereby different genes that target different viral functionalities are delivered into separate cell populations. Model simulations indicate that under some conditions, this idea, which is uniquely applicable to gene therapy and has not been analyzed previously, can dramatically prolong viral suppression and decrease the likelihood of escape. Finally, we study the development of resistance when the gene-modified cells have a proliferative advantage over untreated cells. Simulations demonstrate mixed implications on viral escape, namely, that it is less frequent but that when it does occur, it occurs more rapidly. The presented work provides a basic and general understanding of the key characteristics of gene therapy and their role in the evolution of resistance. Model predictions thus offer guidelines to optimizing therapy for long-term suppression of HIV-1 in patients.

## Results

### Motivating Data

Gene therapy is still a nascent technology; however, there have been a number of studies that serve to motivate our model. Here, we briefly outline the methodology and findings of several studies and discuss how our modeling work was inspired by them.

The first study is a phase I trial in which CD4+ T cells were harvested from five HIV-positive patients, transduced *ex vivo* with a lentiviral vector expressing an antisense RNA targeting HIV, amplified, and then infused back to the patients [5]. The patients were followed for several years, throughout which their immunological function and the persistence of the gene-modified cells were assessed. This trial not only demonstrated long-term survival of these cells *in vivo*, but also showed sustained and statistically significant reductions in the viral load in several patients. However, the modified cells declined in number following the infusion, and persisted at frequencies lower than 1% for most of the trial duration. These findings suggest that the cells are imposing some sort of selective pressure on the virus, although their mechanism of action is currently unclear as gene modification frequencies were too low to account for the observed changes.

As we mentioned earlier, current transduction efficiencies are low, implying that the modified cells must accumulate *in vivo* to reach therapeutic numbers. Such trend has not yet been observed in early-phase anti-HIV trials [24], indicating that the selective advantage of these cells *in vivo* is not sufficiently high. This may be because the engineered genes or their products lose their activity *in vivo*, and/or because the cells' proliferative capacity was impaired during their *ex vivo* manipulation. Current attempts to tackle these issues focus on increasing the vector-copy numbers per cell, and on intensive development of culture systems that better enrich and maintain T cell subsets which display extensive replicative capacity (reviewed in [34], [35]). Encouraging results from two recent studies are also worth noting. In one study, cells modified with zinc-finger nucleases expanded to therapeutic levels and induced substantial clinical effects in a mouse model of HIV infection, thus demonstrating their efficacy and selective advantage [30]. A clinical trial to test this approach in humans is currently underway. In a second study, the long-term (i.e., two years) expression of therapeutic genes in human blood cells *in vivo* was confirmed [36]. Clearly, the enabling technology is yet to mature, but once these barriers are overcome and gene therapy enters the clinic, viral resistance is to become the major concern. This is the starting point to our study, and one of our goals is to understand the implications of a potent gene therapy on viral evolution and what may be done to prolong its therapeutic effects in the presence of a rapidly mutating virus.

Another noteworthy phase I trial is a very recent one, in which CD34+ hematopoietic progenitor cells of four patients were transduced with a lentiviral vector expressing a combination of three unique gene therapies [36]. While combination therapy similar to HAART has been promoted in the gene therapy field as a method for combating viral escape, this is the first trial to put this idea into practice. In this trial, all three therapies were expressed from the same vector, a technique that provides the highest levels of protection in each cell, but also requires significant optimization and is subject to constraints. Given that more combination therapies will likely be developed, we asked how effective such combinations are in maintaining long-term viral suppression. Furthermore, gene therapy opens up a unique opportunity to split such combinations across cells, such that some cells express one therapy and others express another therapy. Such approach may offer an appealing and less technically demanding alternative to current combinatorial approaches. Here, we aim to explore its potential to provide significant improvements in preventing escape.

### Model Overview

**Figure 1:**
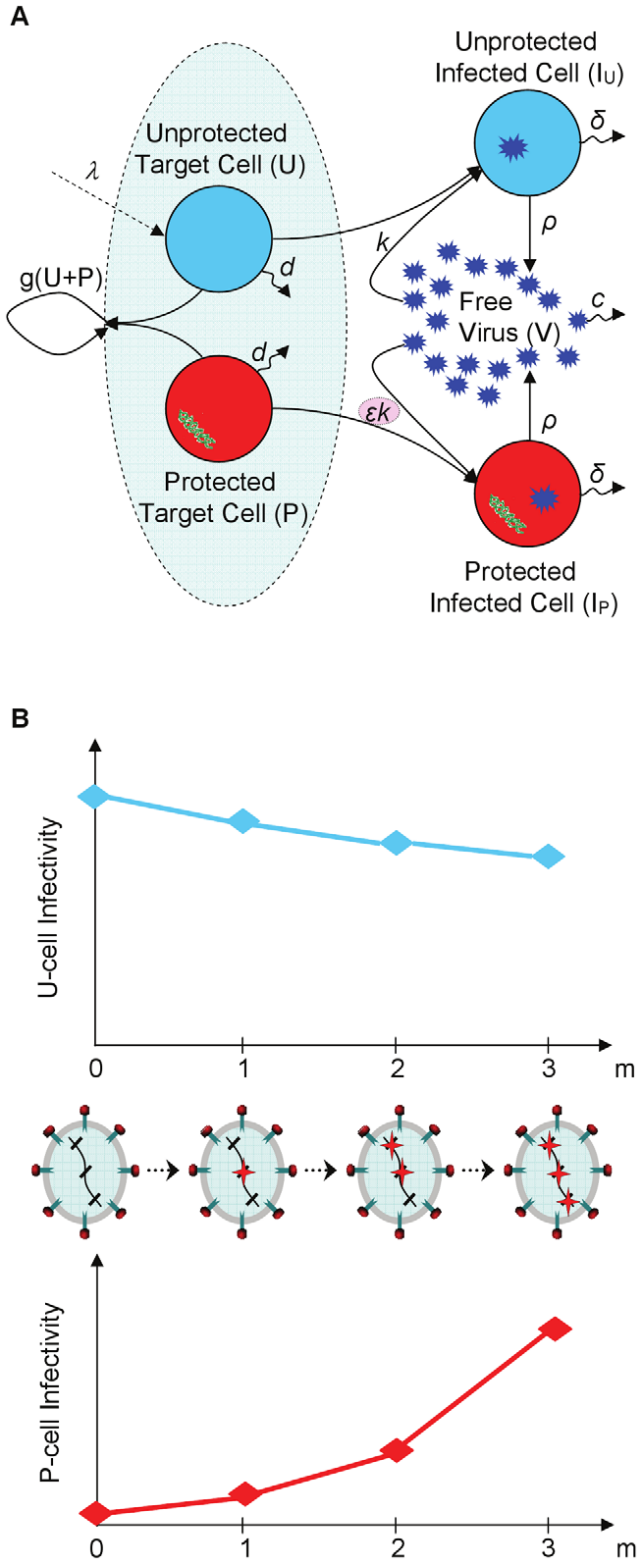
Schematic illustration of infection dynamics under gene therapy and of the development of resistance to it. (A) Diagram of the baseline model, which considers only a single sensitive viral strain. Two types of T cells are considered: gene-modified cells which are protected from infection (shown in red) and non-modified cells that are susceptible to it (shown in blue). The proliferation rate of both cell populations is determined by a saturating function, 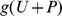, which takes a Michaelis-Menten form. Therapy effects are conveyed via an infectivity inhibition factor (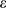) for gene-modified cells. (B) An example of a possible evolutionary path towards the emergence of a highly resistant strain. A genetic barrier 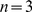 corresponds to a set of three resistance-conferring sites (hashes), where any combination of these sites can be mutated (stars). At each integration step of the simulation, a strain may only accrue a single mutation in one of the non-mutated sites. The degree of resistance is determined by the number of mutations (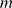), and is manifested as improved infection of P cells (i.e., higher infection rates due to an increase in 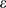). Mutations are also associated with a loss in fitness that negatively affects the ability of these mutants to infect U cells, and that also depends on the number of mutations.

The model consists of two types of susceptible CD4+ T cells: transduced cells that are infused to the patient (protected (P) cells), and naturally occurring cells that were not manipulated (unprotected (U) cells). The overall CD4+ T cell pool is maintained by homeostatic proliferation, which saturates according to Michaelis-Menten kinetics (Figure 1A) [37]. Both cell types are regulated by the homeostatic mechanism in the same manner, and contribute equally to saturation, thus equally competing for presence in the pool. The renewal of susceptible cells is assumed to rely mainly on self-proliferation, with additional minor contribution from the bone marrow, modeled as a constant export of mature cells from the thymus. In this work, we focus on delivery of T cells, as opposed to stem cells, and hence the bone marrow engenders only U cells. HIV infection dynamics follow standard HIV models [16], [17], [38], with the therapy effects manifested as an inhibition of viral infectivity of the P cells.

The model considers a viral population consisting of a sensitive wild-type (WT) strain as well as other strains to which WT may evolve through a series of mutations. There are *n* genomic sites that confer resistance to therapy, and each mutant strain can have any combination of them mutated away from their WT form. Resistance is assumed to gradually intensify with increasing numbers of mutated sites, and it manifests as an improved ability to infect P cells, but is at the same time associated with a fitness cost when infecting U cells (Figure 1B). The model captures all interactions via ordinary differential equations (ODE), but uses a stochastic routine to treat populations at low densities, such as those of newly-emerging species (see the Methods section for details).

### Viral Dynamics in the Absence of Resistance

We start by exploring a restricted baseline model, similar to the model of Lund *et al.* [19], in which resistant strains are absent. We use it to demonstrate the inherent differences between gene therapy and HAART and to quantify the effects of different parameters on the achievable viral suppression levels.

**Figure 2:**
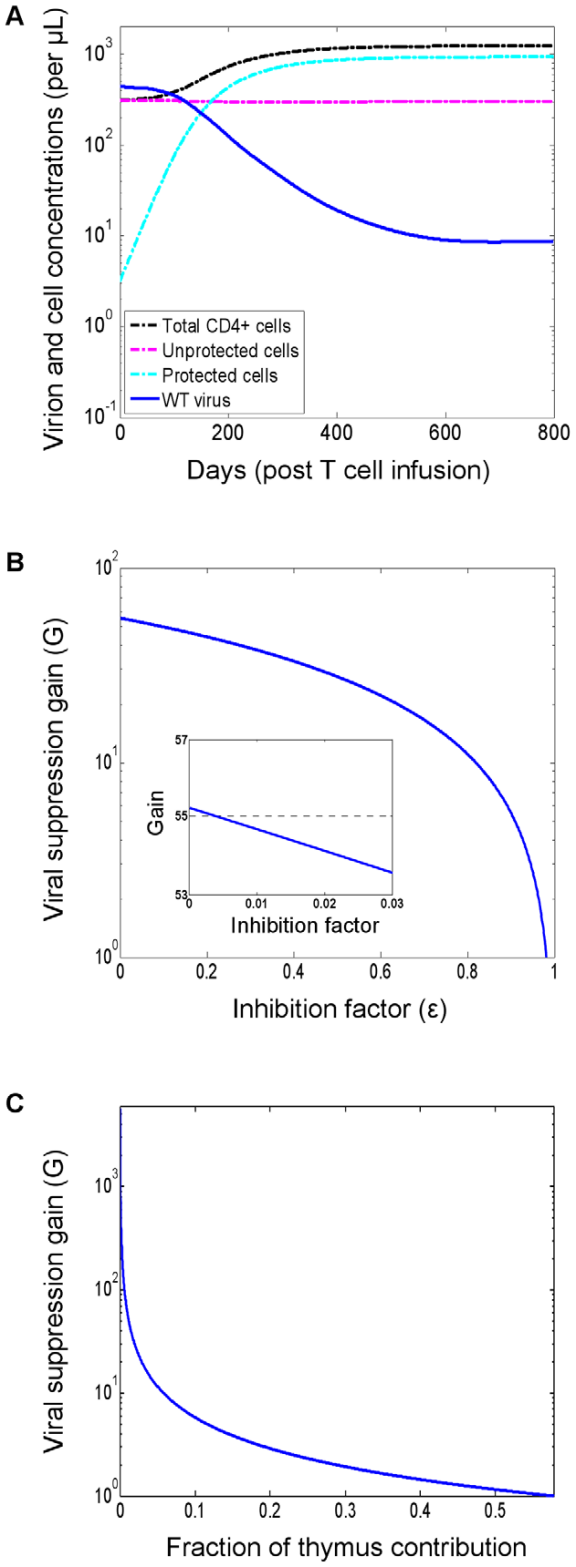
Infection dynamics under gene therapy and the dependence of the viral suppression gain on the inhibition factor and on the thymus contribution. (A) Dynamics in the absence of viral resistance. Simulations were performed with default parameter values (see the Methods section). (B) The suppression gain (*G*) is the ratio between the viral loads in the pre-therapy and post-therapy steady states of the baseline model (see the Methods section for an explicit expression). It depends linearly on the inhibition factor 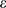 (x-axis) and is depicted on a logarithmic scale. The inset (depicted on a linear scale) demonstrates that decreasing the inhibition factor below a few percent has negligible contribution to viral suppression. The pre-therapy steady state becomes the stable solution when 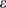 is approximately 0.98 (not shown). All parameters were set to their default values (see the Methods section). (C) The thymus' relative contribution (x-axis) is the fraction of the total T-cell mortality that is replaced by the export of mature cells from the thymus, as opposed to by self-proliferation of the T-cell pool. The suppression gain is inversely proportional to it and diminishes quickly. HAART-like gains may be achievable only for diminutive relative contributions. When the contribution is sufficiently large, no viral reduction is obtained, and the pre-therapy steady state becomes the stable solution (not shown). In order to keep the pre-therapy conditions fixed for all points, the proliferation rates and parameters were adjusted to compensate for a varying thymus input. Other parameters were set to their default values (see the Methods section).

Figure 2A shows *in vivo* dynamics following a P cell infusion, as simulated with default parameter values (see the Methods section). The P cells are shown to expand, thereby impeding the supply of U cells and thwarting viral replication. The establishment of a P cell reservoir brings the virus to a new reduced set point. The P cells' expansion is driven by a selective advantage they possess over U cells, which derives from a reduced susceptibility to infection. The expansion thus slows down as the viral load declines.

In comparison to post-HAART behavior [39], [40], three substantial differences are clear: the heterogeneity of the target-cell population, the slow nature of the response, and the limited reduction in viral load. In particular, HAART blocks viral replication on nearly the entire target-cell population, resulting in a rapid viral decline. This is in contrast to our model, where the U cells experience only a slight decline, and continue to facilitate viral replication, albeit at lower volumes due to their reduced turnover rates. In addition, HAART takes effect promptly and comprehensively, whereas gene therapy induces a gradual depletion of the U-cell supply, fueled by the inherently slow process of P cell accumulation. To summarize, the two approaches exhibit different mechanisms of action: brute-force disruption of viral activity by blocking access to existing resources (HAART) versus draining the supply of those resources in favor of more robust ones (gene therapy). Since gene therapy profoundly re-structures the blood system to render it less susceptible to HIV, it is slow to exert its effects. Yet, only innate changes of this kind can facilitate sustained virus control with limited medical intervention. HAART, on the other hand, is quick to act, but once interrupted, results in immediate viral rebound and thus necessitates a strict lifelong adherence to an elaborate drug regimen.

#### Therapy efficacy

We developed metrics and analysis to quantify the therapy's potency in suppressing the virus. Within this work, we assumed that infectivity is inhibited by a factor of 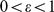. We further assumed that virion production in infected P cells is completely eliminated, which can be realized by inserting additional antiviral genes that target late stages of the infection [4], [41], [42], [43]. Importantly, both infectivity inhibition and progeny suppression contribute similarly to disrupting viral replication [39]; however, the former is essential to the expansion of P cells, whereas the latter does not confer them with any selective advantage. Due to very low dosages of infused cells, progeny suppression alone would only exert a weak and transient effect [21]; however, by providing a selective advantage to modified cells, infectivity inhibition allows the P cells to reach therapeutic levels and, in combination with progeny suppression, provide the latter with the opportunity to further disrupt HIV [21]. This can be thought of as an “ideal” best-case scenario, which elucidates the ultimate potential of infection inhibition. We also compared the ideal case to the baseline case, where virion production is unaffected (Figure 1A), and found that for currently plausible 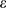 values (a few percent or less [10], [44]), the differences in dynamics were negligible. We thus performed steady-state analysis of the best-case model and found that despite the complex suppression mechanism, the degree of viral reduction depends only on three parameters: the inhibition factor 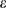, the thymus-input rate 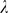, and the infected-cell life span 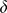 (see the Methods section for explicit expression).

We define the *suppression gain G* to be the ratio between pre-therapy and post-therapy steady-state viral loads, and the expression for this metric (see the Methods section) enables analysis of the system's dependencies on key parameters. Interestingly, *G* depends linearly on 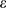, as illustrated in Figure 2B (note that *G* is depicted on a logarithmic scale). It ranges between a maximum of nearly two logs at 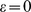 and zero at 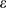 very close to 1. From a therapy design perspective, the important point here is that there is negligible incremental benefit for highly potent inhibition, especially when going below the reasonably achievable levels of a few percent [10], [44] (shown in the inset). This finding echoes previous predictions that were derived from different models [21]. However, as we shall see later, potent inhibition is instrumental in controlling viral resistance and hence should not be overlooked.

In addition, *G* has an inversely-proportional dependence on 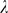 (Figure 2C). In Figure 2C, 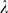 is expressed in terms of the thymus' relative contribution to overall T cell renewal, a measure that is proportional to 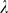. One can see that significant gains are achievable for extremely small thymus contributions, but they quickly diminish up to a point of no viral reduction, in which case the post-therapy set point becomes unstable. This indicates that the bone-marrow contribution can significantly undermine the ability to achieve considerable viral declines. Although its relative contribution is known to decay with age and to account for a small fraction of the T cell renewal in adults, 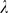 has not been accurately quantified to date, and current estimates range from a tiny fraction to more than one tenth [45], [46], [47], [48]. Quantifying 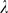 thus constitutes an important step in understanding the potential clinical benefits and limitations of a T-cell based approach. A possible way to circumvent such limitations is by enhancing the P cells proliferative capacity [21], and we will examine it later from the viewpoint of viral resistance.

### Emergence and Fixation of Resistant Mutants

The integration of resistance into our model gives rise to an additional therapy characteristic, namely, a genetic barrier 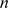, which we define as the number of mutations that the virus must accumulate in order to completely overcome inhibition by P cells (Figure 1B). To preserve the simplicity and tractability of the model, we assumed that a mutant's phenotype is determined by the number of accumulated mutations, but not by their actual identity or location (see the Methods section). In other words, strains that possess different combinations of *m* mutations are phenotypically indistinguishable. Our model limits the mutational effects to modified infection rates (i.e., varying attenuations of WT's infection rate) for two reasons. First, this is the most likely route to escape [10]. Second, it is the most effective one, since once the P cells expand and the increased infection rates allow the virus to infect significant portions of them, only then virion production can exert meaningful effects. As gene therapy involves two cell populations, the model accounts for contradicting effects on both cell types, as follows. On one hand, resistance confers the virus an improved ability to infect P cells, while on the other hand, the accumulation of mutations in key regions of the virus targeted by the therapy can also be associated with a reduction in fitness in the absence of therapy, that is, in U cells [23], [49], [50], [51], [52]. The model assumes that both effects gradually escalate with each additional mutation [10], [50], [52], [53], [54]. Since our model attempts to broadly apply to several techniques, it perceives the genetic barrier as a general property and disregards its specific origins. A large 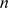 may thus correspond to several inhibitors acting concomitantly, or to a single inhibitor whose interaction with the virus spans a large domain [10]. A similar interpretation applies to the inhibition factor 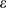.

**Figure 3:**
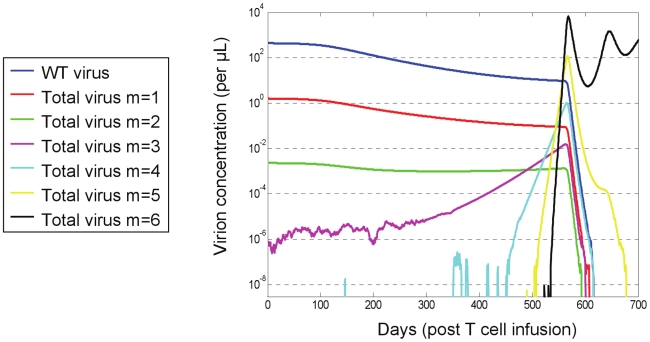
Dynamics of resistance evolution in a typical hybrid stochastic-deterministic simulation. Following T cell infusion, a decline in wild-type (WT) virus and pre-existing mutants is accompanied by the emergence of fitter and highly resistant mutants. Mutant strains are aggregated according to the number of resistance mutations they carry (*m*), which determines their resistance phenotype. Carrying more mutations renders a strain more resistant, with the most resistant strain carrying *n* = 6 mutations. Stochastic effects are observed at very low densities, where advantageous mutations experience drifts before accumulating to critical levels which enable their consistent expansion. Populations whose density decreases below a pre-defined threshold (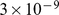 entities per µL) are considered extinct until they resurface. In this simulation, the total mutant density reaches WT density at approximately 550 days after infusion, marking the fixation of the resistant strain. Simulation was performed with default parameter values (see the Methods section).

We performed stochastic simulations with our model and aggregated the mutants' densities according to number of mutations, such that all strains with 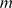 mutations (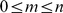) count as one species. Figure 3 shows the outcome of a typical simulation of the stochastic trajectory when 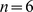. It demonstrates an accumulation of increasingly fitter mutants at the expense of less competent pre-existing (e.g., 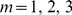) and early-appearing mutants (e.g., 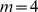). Mutants that possess higher numbers of mutations (e.g., 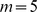) are increasingly more competent, and eventually reach levels that give rise to even fitter strains (e.g., 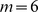), which soon outgrow them. In the presence of highly resistant strains, the P cells lose their advantage and decline (not shown). Note that the decline of pre-existing mutants with one or two mutations is a consequence of both the drop in the WT viral population, which continuously feeds the mutants populations, and of their susceptibility to therapy. However, the decline is slow, thereby constituting a major obstacle for gene therapy, as it fuels progressive mutation accumulation and expedites the emergence of non-existing resistant strains. In contrast, under HAART, pre-existing strains swiftly drop to very low levels, and while still present, their mutation into more resistant strains is severely confined by their small absolute numbers.

The stochasticity that arises from the random effects that dominate at small population sizes can be seen for existing mutants with three mutations and for newly emerging mutants. New advantageous mutations occur at random and may drift away before reaching critical levels, thereby delaying emergence in comparison to fully-deterministic trajectories [18]. As delays build up along the evolutionary process, larger 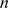
's are progressively associated with increasingly varying fixation times of highly resistant strains (see Figure S1). As a result of this stochasticity, it is striking that two “identical” patients (i.e., infected with the “same” virus) can experience remarkably different clinical outcomes.

**Figure 4:**
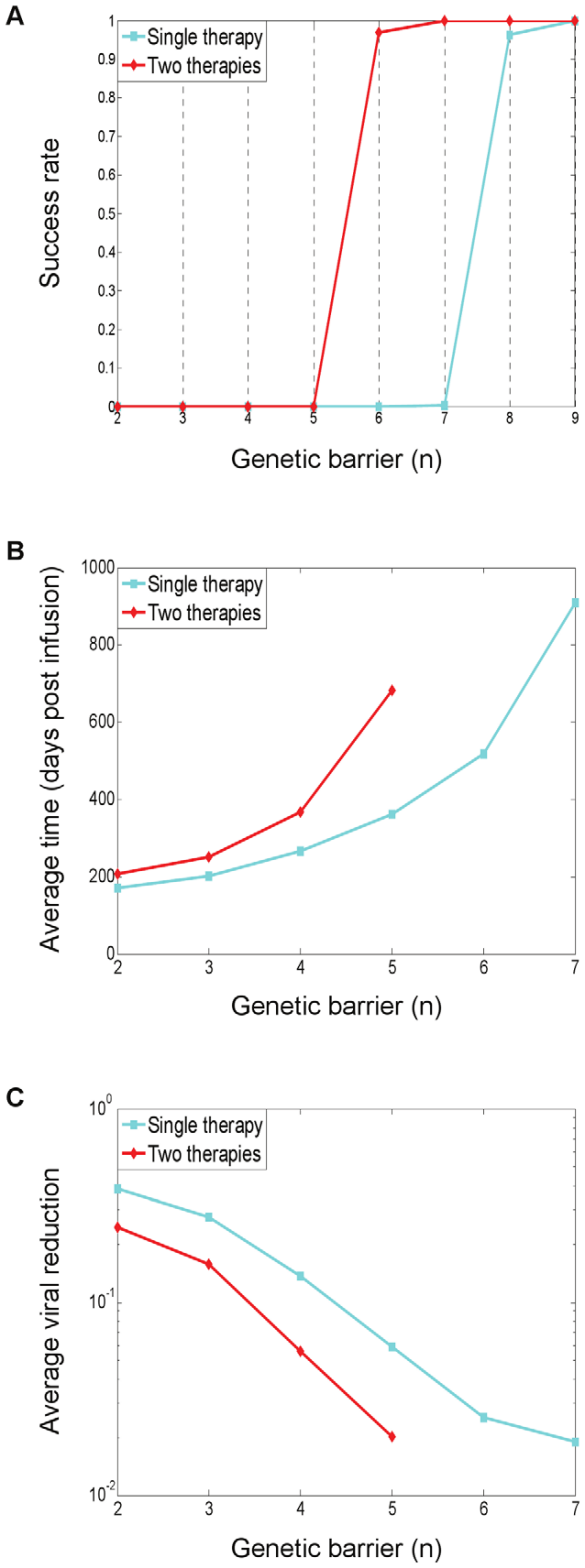
Quantitative evaluation of treatment strategies investigated in this study. Each point summarizes the average outcome of 500 simulations with default parameter values (see the Methods section). The blue curves correspond to a standard use of a single gene therapy (discussed in subsection “Emergence and Fixation of Resistant Mutants”). The red curves correspond to a combination-therapy strategy whereby the P cells are divided into two sub-populations, each protected by a distinct set of genes (discussed in subsection “Divide and Conquer – an Effective Strategy to Combat Resistance”). (A) Effects of increasingly larger genetic barriers *n* (x-axis) on the fraction of successful treatments (y-axis), featuring a threshold-like behavior. A treatment is considered successful if mutant fixation did not occur within four years after its initiation at day 0. (B) Effects of increasingly larger genetic barriers *n* (x-axis) on the average time to fixation (y-axis), which is the time required for the resistant strains to reach 50% of the viral population. Data are depicted only in cases where the success rates are below 0.9, representing at least 50 fixation events per point. (C) Effects of increasingly larger genetic barriers *n* (x-axis) on the average viral reduction at the time of fixation (y-axis), depicted when success rates are below 0.9. The viral reduction is the ratio between the viral loads at fixation time and at day 0. Large genetic barriers are needed in order for the therapy to reach its set point before resistance emerges. (A,B,C) Viral suppression under the two-therapy regimen is gradually prolonged throughout the entire barrier range, and displays a dramatic advantage at 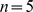 (B). Success rates and viral reduction are also improved (A,C).

As a measure of the treatment's efficacy, we computed the fixation time, defined as the time required for the resistant strains to reach 50% of the viral population. Treatment was considered successful if fixation had not occurred within four years of its initiation. Figure 4A–4C provides an overview of the three figures of merit by which we evaluate a treatment. Each point in the plots summarizes the results of 500 simulations with default parameter values (see the Methods section). The blue curves correspond to a standard application of gene therapy, as discussed above, whereas the red curves correspond to a more advanced strategy and will be discussed in a later subsection. At this point, we limit discussion to the blue curves. Figure 4A shows how the fraction of successful treatments, called the success rate, varies with the genetic barrier *n*. Interestingly, success rates exhibit a threshold-like behavior, which was found to be typical with many other parameter choices (not shown). Such an effect becomes important when one considers combining several gene-based inhibitors within a P cell as a means of increasing 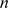. If a therapy is near the threshold, the addition of an inhibitor can make a dramatic difference in its efficacy. Figure 4B shows average fixation times for all 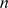
's for which therapy success rates were below 0.9 (see Figure 4A). As expected, larger 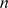
's result, on average, in prolonged viral suppression. Figure 4C shows the average of the corresponding viral load reductions (i.e., the inverses of the suppression gain) obtained at the fixation time. One can see that the average viral reduction keeps declining until 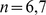, reflecting the fact that for 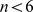, resistance emerged before therapy has reached its steady state in the absence of resistance. This implies that under our default parameter choices, even considerable genetic barriers (e.g., 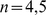) do not suffice to allow therapy to reach its full suppression potential before resistance emerges.

Finally, we wish to stress that the inhibition factor 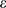 is assumed to be independent of 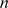 and thus was kept fixed throughout simulations. Specifically, a larger 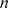 does not imply a stronger inhibition, even when obtained through insertion of additional genes. The dynamics under gradually increasing inhibition can be readily obtained using similar simulations.

### Replication Fitness over a Heterogeneous Target Environment

The notion of *replication fitness* has long been used for understanding HIV's evolution under HAART [55], [56], [57], [58]. It is a model-derived measure of a strain's ability to expand over time in a given environment, and provides a tool for understanding and predicting clinical outcomes. This measure, however, was derived from HAART models and does not apply to gene therapy, whereby HIV is faced with a mixed cell composition and may display different replication traits within each cell subpopulation. We used our model to extend this notion to the case of a heterogeneous target-cell environment (see the Methods section and Text S1 for derivation). The replication fitness (*F*) of a strain 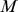 is captured by the following expression:
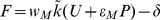
(1)where *U* and *P* are the two cell-type densities, 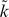 is a generalized infection rate constant (see the Methods section), 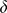 is the infected cells' death rate, 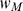 is a replicative fitness cost associated with mutating, and 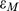 is the infectivity-attenuation factor. The parameters 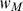 and 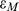 summarize the cost and benefit involved in viral escape, and are determined by the number of mutations in strain 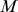, as well as by the therapy's potency (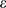) and genetic barrier (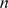) (see the Methods section and Figure 1B). The parameters 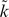 and 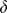, in contrast, are unaffected by viral evolution, and so distinct viral strains feature different 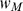 and 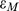, which, when weighted through Eq. (1), yield an overall replication fitness. Importantly, *U* and *P* are time dependent, and so *F* varies as therapy progresses. Yet, changes to *U* are minor in our model, and *P* transiently increases until it saturates.

Eq. (1) encapsulates the key determinants of viral resistance and provides insights into therapeutic design tradeoffs. We illustrate this point with a simple example. Consider a potent therapy that inhibits WT's infectivity with 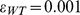 (Figure 1A), and suppose that the U and P cell densities both equal 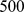 cells/µL. The WT virus' fitness is then given by 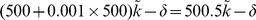. Consider two strategies to weaken the virus: applying a tenfold-stronger infectivity inhibition (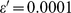) or interrupting its normal function such that it experiences a modest fitness loss of 5% (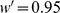) but the same inhibition as before (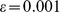). The corresponding changes to the replication fitness amount to 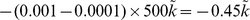 in the first case, compared to a more favorable 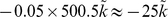 in the latter case. Even starting with 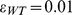 and applying a 100-fold decrease (yielding the same 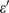) attains only a decrease of 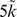 in the first case, which is somewhat more comparable to the losses from the moderate 5% replicative cost. Clearly, realistic tradeoffs depend on the U and P densities as well as on simultaneous changes in all parameter values, but this example stresses the different impact of 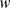 and 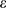 on controlling viral replication. It shows that viral fitness in the U cell pool plays an important role in restraining replication, provided that mutations are associated with a replicative cost, and that powerful protection in P cells has a modest contribution in comparison. Next, we use simulations to show that modest fitness penalties can impede resistance as effectively as major increases in potency.

### Effects of Fitness Cost and Inhibition Potency

**Figure 5:**
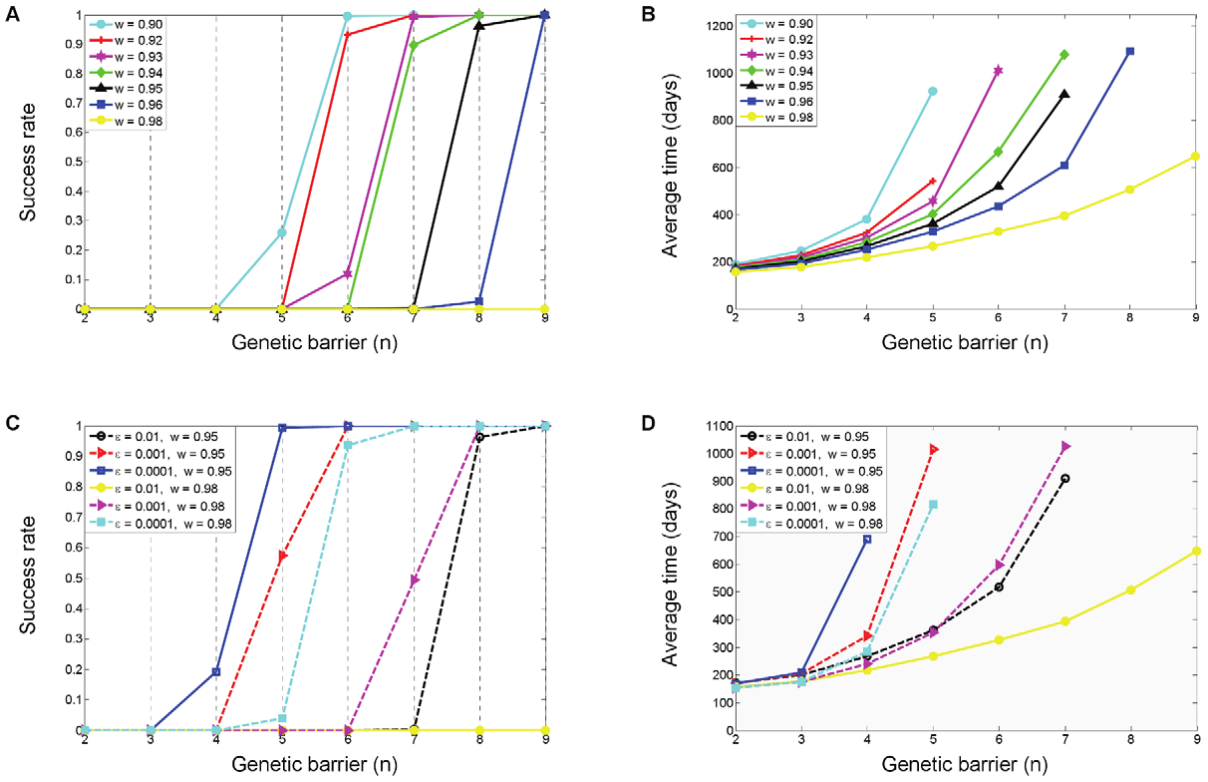
Effects of altering the fitness cost and the inhibition potency on therapy efficacy. Success rates (fractions of successful treatments) and average fixation times are shown as functions of the genetic barrier 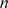 (x-axis) for a range of mutational fitness costs 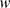 (A,B) and for a range of inhibition factors 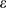 (C,D). The data summarize the outcomes of 500 simulation runs per each parameter set, similar to Figure 4. In each panel, different colors are used to depict different parameter values, with black depicting the default case. The plots demonstrate that increasing the fitness cost (lowering 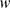) and decreasing the inhibition factor 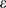 both result in prolonged viral suppression. This is manifested in improved success rates and in delayed resistance emergence. Panels C and D depict the effects of varying the inhibition factor for two distinct fitness costs (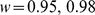), illustrating the tradeoff between the two factors. It can be seen that a tenfold increase in inhibition (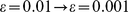, 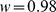) is as effective as a slight decrease in fitness cost (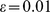, 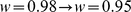). Panels C and D also point out that minor fitness costs (e.g., 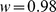) must be compensated by powerful inhibition (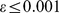) in order to attain long-term suppression with potentially achievable genetic barriers (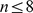).

To further explore the roles of mutational fitness cost and inhibition potency in controlling resistance, we simulated the model over a wide range of parameters. We first varied the average fitness cost that the virus incurs with each additional mutation (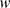), while fixing all other parameters at their default values. Figure 5A–5B shows success-rate and fixation-time graphs, spanning a range of fitness costs. As expected, greater fitness penalties hamper escape and shift the “success threshold” towards lower 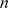
's. Increased penalties also delay therapy failure (fixation time), allowing for greater viral reductions (not shown). One can also see that it is essential for gene therapy to target viral functionalities for which resistant mutations incur non-negligible costs (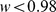).

The effects of altering the inhibition factor 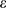 are shown in Figure 5C–5D for two fitness costs. Here, the inhibition was amplified tenfold each time, resulting in meaningful improvements. It can be seen that when mutational fitness costs are minor (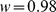), highly potent inhibition is needed to effectively hamper resistance, as this is the only way to restrain the virus. The tradeoffs between the two factors are exemplified by the two sets of nearly overlapping curves (shown in dashed lines), which correspond to a tenfold increase in inhibition coupled with a slight decrease in fitness cost.

While mutational fitness cost for viral replication in the U cell pool appears to dominate resistance dynamics, our simulations also indicate that powerful inhibition can in fact control the virus, and constitutes an important design criterion. It takes effect by weakening early mutants, such that for a given 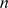, more mutations are needed to reach a sufficiently fit virus. One means to achieve increased potency may be through combination therapy, provided that the individual effects are multiplicative. Nonetheless, to the best of our knowledge, the incremental contributions of single therapies within an ensemble have not been determined to date.

### Divide and Conquer – An Effective Strategy to Combat Resistance

Combination therapy has traditionally been the treatment of choice against the rapidly mutating HIV-1. Simultaneous targeting of several functional domains slows down resistance by concurrently increasing the genetic barrier and strongly suppressing replication. Gene therapy adopted this principle [12], [13], [41], but more importantly, opened the door to a new combination strategy that has hitherto been infeasible, that is, of combining targets across cell populations as opposed to within individual cells.

*Ex vivo* gene transduction provides the clinician with control over the destination of delivered drugs, which, in turn, enables the infusion and *in vivo* expansion of distinct P cell pools, each containing different inhibitors that target distinct viral functionalities. In this setting, a strain that resists one inhibitor is confined to replicate only on a fraction of the P cell population, and is still suppressed within the rest of it until it acquires additional mutations. The idea, then, is to limit the resources available to the evolving virus by forming sub-populations of P cells.

We illustrate this principle and explore its potential using a computational model (see the Methods section and Text S1). Since this strategy can be used on top of any gene therapy technique, it represents an additional mechanism to combat resistance. We thus seek to quantify its added value compared to using homogeneous protection. Our model considers two gene therapies, P1 and P2, targeting distinct viral functions such that cross-resistance between them is excluded. This may apply, for example, to binding and/or fusion inhibitors (P1) combined with integrase and/or reverse-transcriptase inhibitors (P2). Both therapies are modeled as equally powerful - they display the same 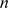 and same 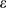, and each may correspond to one or to several concurrent inhibitors. When introduced into two cell populations, they divide the P cells into two smaller sub-populations and give rise to a complex quasispecies environment with viral strains that display a range of resistance levels to one or both therapies. The baseline case, as captured by the previously discussed model, corresponds to use of just one of these therapies.

The resulting average fixation times and success rates were compared to their single-therapy counterparts (Figure 4A–4B). We found that fixation times improved gradually (but modestly) throughout the entire range, until a dramatic increase in favor of therapy combination took place at 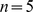 (Figure 4B). The same effect is manifested as a meaningful shift in the success-rate curve in favor of combination therapy - it suffices to use two therapies with 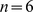 as opposed to 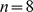, which is required for a successful stand-alone approach (Figure 4A). Figure 4 pertains to simulations performed with default parameter values, but we observed the same trend for a wide range of other parameter values, with some variation in the position (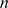) of the dramatic shift (data not shown). Simulations with three distinct therapies showed further improved gains at all levels, as expected (data not shown). We conclude from our findings that this approach is powerful when used judiciously within the “right” 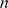 range.

### To Split or Not to Split – Comparison of Combinatorial Strategies

Another interesting question is how the added value of splitting therapies across cells compares with the added value of increasing the genetic barrier. This question is of significance to gene therapy, where large 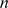
's are necessary to guarantee long-term suppression (Figure 4). Large barriers will likely be accomplished through combinatorial approaches (within each cell), which in turn, are associated with technical and physiological challenges [59], [60] that may consequently limit the achievable barriers. This is especially relevant to RNAi therapy, which is characterized by low individual barriers and strongly relies on combinatorial approaches, but at the same time, faces serious obstacles associated with multiple payloads [61]. In light of these challenges, splitting therapies may offer an alternative to pursuing large multi-component payloads.

**Figure 6:**
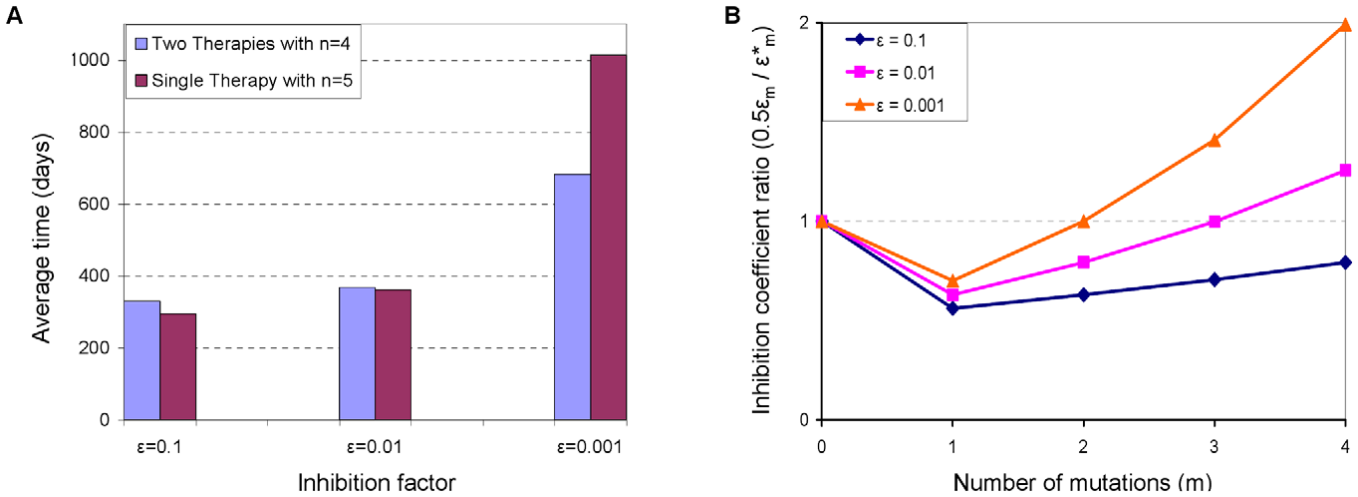
Efficacies of different combinatorial gene therapy strategies. Two strategies are considered: protecting the entire P cell population with a single gene-based inhibitor whose genetic barrier is 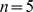, and dividing the P cell population into two sub-populations, such that each one is protected by a different gene-based inhibitor whose genetic barrier is 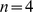. (A) The efficacies of the two strategies are compared in terms of their resultant average fixation times, for three different inhibition factors (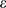). All other parameters are set to their default values (see the Methods section). The different relations between fixation times that are observed for different 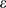 values suggest that none of the strategies is universally advantageous. Data are based on the outcomes of 500 simulation runs per each parameter set, similar to Figure 4. (B) The ratio between the approximate inhibition levels that each strategy exerts on a given strain is depicted (y-axis) as a function of the number of mutations (*m*) a strain possesses (x-axis), for each of the three considered 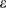 values. The ratio is greater than one when the single-therapy strategy inhibits a strain more potently than the two-therapy strategy, and vice versa. It can be seen that the ratios correlate with the advantageous strategy, that is, larger ratios pertain to improved performance of the single-therapy strategy.

We used our model to answer the following question: is it more beneficial to use two therapies whose genetic barrier is 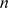 or to invest the effort to design one therapy with an enhanced barrier 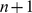
? We show that there is not a definitive answer to this question, as illustrated in Figure 6A for 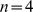. Average fixation times under both strategies are depicted for a range of inhibition factors 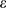, where each case resulted in a different answer. The “splitting strategy” appears to perform slightly better in the presence of a relatively weak therapy (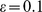), but loses its advantage under more potent regimens. Similar situations were observed for other 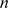 values (data not shown).

We interpret our ambiguous results by examining the viral replication fitness (Eq. (1)) under both conditions. Consider a resistant strain *M* whose replication fitness equals 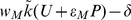. When two therapies are split across cells, one can think of it as downsizing the pool of P cells susceptible to *M* by a factor of two, corresponding to a replication fitness of 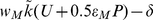. On the other hand, an enhanced genetic barrier typically renders the mutants less resistant than before the enhancement, as they now need to overcome additional inhibitory mechanisms. In our model, this is reflected in a smaller inhibition factor 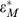, which should be compared to 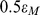. An important point here is that in practice, the extent of decrease from 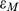 to 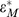 is case-dependent. Since no pertaining data are currently available, we determine 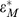 based on simple functional relations between the inhibition factor and the number of mutations (see the Methods section). Our results are therefore model-specific, yet they support our main point that the answer is nontrivial and that strategies should be compared on a case basis.

We further linked our findings to the suggested interpretation by computing the ratios 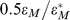 when 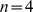. In our model, resistance is a function of the number of mutations, giving rise to five distinct ratios 
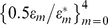
 for each case considered, as depicted in Figure 6B. The considered cases differ in their initial inhibition potency (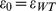), which determines all intermediate inhibition factors. The correlation between the ratios and the advantageous strategy can be readily observed – the more potent a therapy is (i.e., smaller 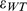), the sooner the ratio curve crosses one, at which point the single-therapy strategy imposes stronger inhibition. For example, in the weakest therapy case (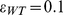), the curve is entirely below one, which means that the “splitting strategy” presents the virus with harsher conditions throughout its escape route. In the other extreme case (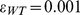), a single therapy exerts stronger inhibition fairly early in the evolutionary process, even on pre-existing mutants. The middle case shows mixed effects, which balance out to yield similar fixation times.

In our analysis, we assumed that increasing 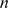 has no implications for therapy potency, or in other words, that 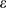 is independent of 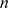. However, when an increase in 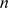 is achieved by the insertion of additional genes into each P cell, it may be reasonable to assume that 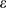 concomitantly becomes smaller. If we further assume that 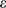 decreased tenfold during the transition to a larger 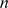, then our simulations indicate that fixation times are consistently longer for the single therapy regimen (Figure 6A). As we already emphasized, this might be a model-specific prediction that may not hold true under other conditions, but we can certainly state that a simultaneous decrease in 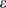 renders the single-therapy strategy more powerful than before. Finally, we stress that such scenario-specific results stand in contrast to the comparison we made earlier, where we found that splitting equally-powerful therapies across cell populations is always advantageous over using one of them alone (Figure 4).

### Effects of Proliferation Enhancement

As we showed earlier, the potential reductions in viral load under gene therapy are limited in comparison to HAART (Figure 2C). Methods to enhance the selective advantage of P cells by extending their proliferation capacity are being explored, as a means of boosting their expansion, such that lower viral set points could be attained. Proposed techniques include expression of stimulatory interleukins, microRNAs, and telomerase reverse transcriptase (hTERT) [34], [62], [63], [64], [65]. While each technique carries the risk of uncheck proliferation that could result in cancer [66], engineering additional safety controls could eventually solve this problem [34]. We chose to consider the idea of proliferation enhancement even though the underlying technology is not fully developed yet, so that we can better understand its impact on the emergence of resistance.

**Figure 7:**
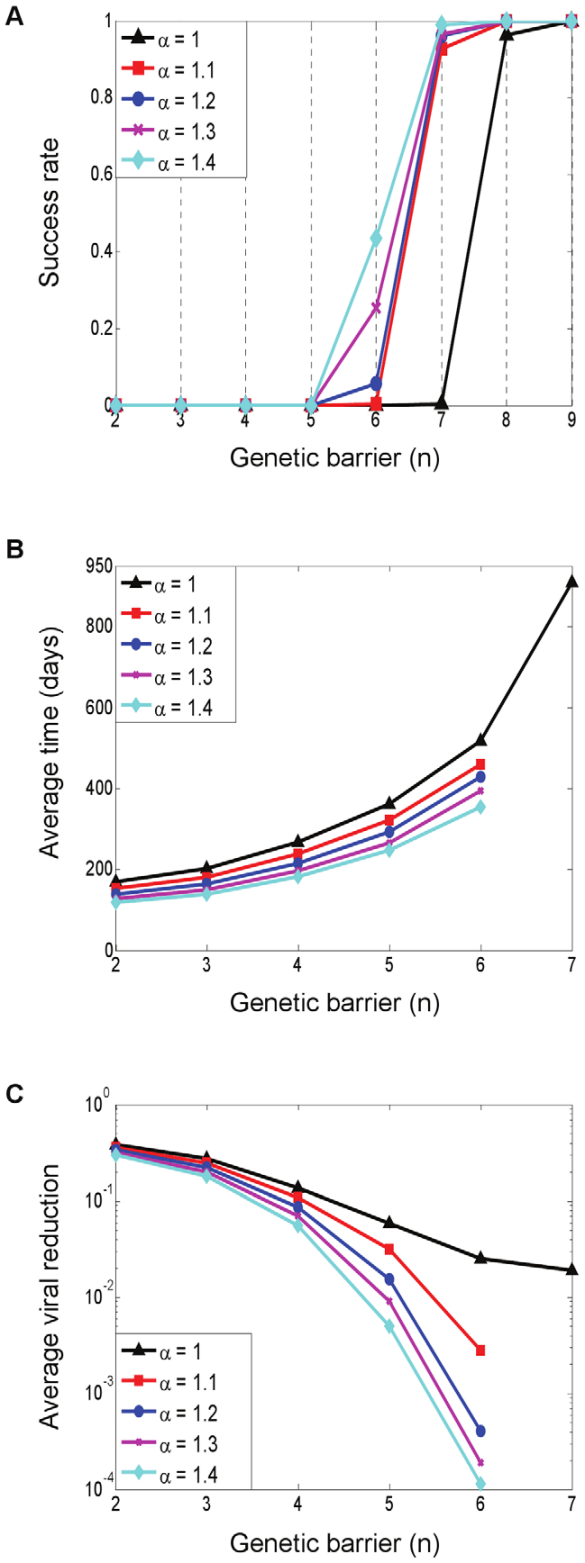
Effects of proliferation enhancement. Success rates (A), average fixation times (B), and average viral reductions (C) are shown as functions of the genetic barrier 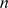 (x-axis) for a range of acceleration factors (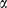). A factor 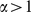 stands for a constant boost (of 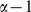 percent) to the proliferation rate of P cells in comparison to the proliferation rate of U cells. Different colors depict different 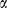 values, with black depicting the default case (i.e., no enhancement). Each point summarizes the outcomes of 500 simulation runs with default parameter values, similar to Figure 4. Success rates consistently improve as a consequence of proliferation enhancement (A), but when the virus escapes, fixation is generally expedited (B). Major improvements in viral reductions take place before fixation occurs (C).

The potential gains from proliferation enhancement were previously illustrated by von Laer *et al.*'s modeling study [21], and our baseline model displays similar trends of reduction in the viral load. Since latently infected cells are precluded from the model, a sufficiently powerful enhancement can in fact eradicate the virus. It also expedites P cell accumulation, thereby intensifying the selective pressure on the virus soon after therapy begins, but at the same time accelerating viral decline (see Figure S2). Figure 7A–7C shows the outcomes of simulations of viral evolution under this strategy, with a range of improved proliferative abilities and with default parameter values. We modeled enhanced proliferation as a constant percentage increase in the proliferation rate of P cells with respect to U cells, represented by a factor 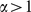 (see the Methods section). We found that enhancing proliferation consistently expedited the fixation of resistant strains and, as explained below, seemingly paradoxically improved the success rates. Overall, the improvements are modest, and gains diminish with increasing 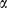. The major therapeutic benefit of this approach is in improved viral reduction before resistance emerged, despite the faster emergence (Figure 7C). In particular, improvements become major for large 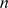
's, amounting to one to two logs. Note that under such conditions, early-appearing mutants that are weakly resistant to therapy may reach 50% of the viral population at very low densities sometime during WT's decline to extinction, albeit without expanding much further. We therefore modified the definition of fixation to exclude cases where the entire viral population continues to circulate well below detectable levels (e.g., 0.01/µL).

The shortened fixation times are not surprising and reflect a combined influence of acceleration in both WT's decline and P-cell expansion. As P cells accumulate, the selective pressure on the virus builds up and fuels the expansion of resistant strains. P cell levels are also higher, which further assists those strains. In light of these factors, it is rather surprising that success rates gradually improved with further enhancement (Figure 7A). We attribute this result to a strong counteractive effect of the drastic viral decline and extinction, which cuts down the *de novo* generation of highly resistant strains. It is also worth noting that we further explored resistance dynamics when proliferation is impaired (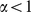), as that may reflect current conditions in gene therapy trials. Therapy effects on the entire viral population are weakened and slowed down in this case, and so, as expected, lesser gains from therapy are associated with weaker and delayed resistance (data not shown).

The effects of proliferation enhancement resemble those of the intensification of traditional anti-HIV drugs [16], and, in fact, the similarity to post-HAART dynamics manifests itself in another way – it allows the P cells to “take over” the immune system. This is because sufficiently intense proliferation renders them advantageous even in the absence of HIV, allowing them to populate the system at the expense of a dwindling U cell reservoir (Figure S2). Fitter P cells occupy bigger portions of the immune system and bring it closer to HAART's homogenous conditions. To summarize, in the absence of resistance, proliferation enhancement constitutes an important step towards achieving HAART-like performance. However, when resistance is accounted for, this strategy may be associated with adverse effects and may prove beneficial under limited conditions.

Table 1 summarizes each of the above-discussed strategies along with their impact on treatment outcomes and their parametric tradeoffs. The first three columns pertain to the qualitative changes in the figures of merit introduced earlier (Figure 4), where the success threshold (first column) stands for the minimal 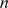 for which success rates exceed 0.9. The fourth column quantifies the changes in the success threshold that correspond to the specified parameter deviations from the default case.

**Table 1:** Influence of strategies for delaying escape on the average treatment outcome.

Strategy	Success threshold (*n*)	Average fixation time	Average viral load at fixation	Change in threshold from default case
Increasing mutational fitness cost (*w*↓)	↓	↑	↓	*w*: 0.95→0.92→*n*: 8→6
Increasing inhibition potency (*ε*↓)	↓	↑	↓	*ε*: 0.01→0.001→*n*: 8→6
Combining therapies across cells	↓	↑	↓	Two therapies→*n*: 8→6
Enhancing proliferation (*α*↑)	↓	↓	↓*	*α*: 1→1.1–1.4→*n*: 8→7

*The improvements in viral declines by fixation time are major, and significantly exceed those achievable under all other strategies.

## Discussion

We developed a computational model of HIV-1's evolutionary dynamics in the presence of gene therapy to study its long-term efficacy against the virus. We used the model to quantify the contributions of key therapy-design parameters and different treatment strategies to the suppression of viral resistance. We find that when judiciously designed, gene therapy has the potential to provide long-term suppression of HIV-1, but meeting this goal requires highly powerful and robust antiviral genes, which may not be currently available. In particular, large barriers, ranging from 5 to 9 (depending on therapy parameters), are needed in order to guarantee that viral suppression below a few percent of pre-therapy loads lasts at least four years. In addition, gene therapy is characterized by very slow dynamics in comparison to HAART, as a consequence of its inherently different mechanism of action. In particular, it can take about two years to reach a new viral set point (Figure 2A). This, in turn, makes a long-lasting resistance control even more critical, if one aims to fully realize the therapy's clinical benefits. We find that delaying resistance emergence merely to the point of reaching a new steady-state viral set point still requires fairly large genetic barriers, which are only slightly smaller than those required for a four-year viral control.

We find that two controllable parameters play a key role in delaying emergence of resistance – the replicative fitness cost associated with mutation (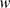) and the therapy's inhibition factor (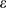). Our results demonstrate that intensifying both factors can severely impair the competence of mutants, and thereby offset the effects of a significant mutational influx of newly emerging strains (Figure 5). Our analysis stressed the difference between the ways by which the two factors 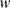 and 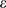 take effect, where the mutational fitness cost acts primarily by hindering viral replication in U cells (Figure 1B) and the inhibition potency serves to hinder the virus as it replicates in P cells. This brings into attention a major difference between gene and traditional therapies, namely that gene therapy leaves a major fraction of the target cells unprotected. As it turns out, one can harness this apparent weakness to improve virus control. Our model predictions support this by showing that small relative decreases in the average fitness cost per mutation dramatically improve success rates and delay resistance emergence (Figure 5). The fitness of mutants in the U cell reservoir, thus, plays an important role here, and targeting highly conserved regions may prove to be particularly beneficial for gene therapy. These results also stress the importance of assessing the fitness of resistant mutants on cultures of unprotected cells as part of a therapy's evaluation.

Our model predicts that sufficiently potent inhibition also provides a powerful means for preventing the development of resistance. However, major amplifications in potency are required in order to achieve benefits that are comparable to those attainable by minor relative changes in fitness cost (Figure 5). For example, we find that 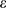
's as low as 10^−4^ can successfully hamper resistance when combined with a barrier 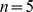. Whether such 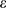
's are practical or not is yet to be determined. Combination therapy, possibly involving different gene-based techniques, may provide a viable approach to achieving this goal, and may simultaneously feature large 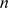
's [4], [14]. Nonetheless, its promise depends on the synergistic interaction between multiple concurrent agents. For example, potent inhibition may be achieved when their joint potency (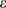) amounts to the product of their individual 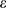
's, rather than to their sum. Importantly, such cumulative effects have not yet been quantified or validated in the context of gene therapy. Note also that the importance of highly potent inhibition in preventing resistance stands in contrast to its negligible contribution in reducing the sensitive virus load (Figure 2B).

The methods presented above are conventional approaches to enhancing an antiretroviral treatment's efficacy, as applied to gene therapy. However, there are supplementary, less obvious, ways to boost gene therapy's performance, which are not possible with HAART. One such method is to combine multiple therapies *across* cells such that the P cells form several sub-populations, where each population is susceptible to different viral strains. We illustrated this novel principle through simulations of an abstracted model, which considers several distinct and equally powerful therapies. Our results demonstrate that dramatic improvement in success rates as well as in fixation times can be obtained in this manner (Figure 4). However, these occur only within a certain window of genetic barriers. When applied outside this window, improvements are modest, albeit increasing with increasing number of therapies. One must therefore reliably characterize the individual therapies before attempting such a strategy.

It is also interesting to compare the improvements obtained by splitting therapies to those obtained by increasing the genetic barrier of a single therapy. We find that the “winning” strategy varies, depending on the therapy parameters and on the resistance spectrum of intermediate mutants (Figure 6). The comparison result particularly depends on 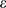, and on whether it decreases while 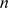 increases. It is reasonable to expect that 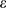 would decrease when 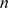 is extended by an insertion of additional therapeutic genes, as long as they exert their effects via distinct independent pathways [13], [14], [41]. In this case, our model predicts that a single improved therapy will perform at least as well as a two-therapy approach. If this is not the case, it is difficult to predict the overall effect on 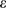 and which strategy performs better. An example of the latter case is a combination of RNAi targets, which is constrained by a maximum tolerable level of siRNA molecules so as to avoid toxicity [67] and competition for cellular resources [68]. It is possible that overall inhibition power may not decrease or may even increase under such constraints, especially when numerous targets are involved. Although combination gene therapy has been extensively studied via experiments over the past few years, we are unaware of any quantitative results that can be used within our model. Nonetheless, our analysis points out the key parameters which need to be quantified in order to compare the two options, namely, the therapies' entire spectrum of inhibition potencies.

Gene therapy pressures the virus indirectly by way of a slow P cell expansion, and its limitations derive from the persistence of U cells. It has been argued that it could be improved by boosting proliferation of P cells, such that they outcompete U cells [42]. We explored the implications of such strategy on resistance dynamics and observed mixed effects on clinical outcomes: the likelihood of viral escape is decreased for a narrow range of genetic barriers, but when escape does occur, it occurs more rapidly than without enhancement (Figure 7). Overall, both effects are moderate, even for increases of 30%–40% in proliferative activity. In light of our results, it is worth noting that this technique is appealing for other reasons. It induces rapid viral decline, powerfully suppresses WT virus, and facilitates an extensive re-population of the immune system with P cells. These major improvements may be associated with further clinical advantages that are not captured by our model, such as the recovery of the lymphocyte homeostasis mechanism, which is believed to be impaired in the presence of high HIV load [47], [69], [70].

It is also worthwhile to consider alternative ways for gene therapy to overcome its limitations. Resorting to hematopoietic stem cells may not suffice, as simulations we conducted show that when the infused fraction is small, both approaches are confined by the same factors and exhibit similar dynamics and efficacies (data not shown). Only when large fractions are infused, can meaningful improvements over T-cell-based methods be obtained (see Figure S3). This is concerning in light of results of recent clinical trials which indicate that current delivery efficiencies reach several percent at best [4], [41], [71]. A potentially promising way to overcome this limitation is by including a selectable genetic marker in the antiviral payload that renders the P cells resistant to a chemotherapeutic agent, to which stem cells are normally sensitive. It thereby allows for their chemotherapeutic selection and expansion *in vivo*. Recent progress in this direction is encouraging, as substantial selective expansions were observed in large animal models that were treated with such drugs [72], [73], [74], suggesting that these may be used with humans [36], [74]. Another option is to ablate the immune system to make space for the P cells, and despite its associated risks, is it currently applied in a number of ongoing clinical trials [75]. From a modeling perspective, these approaches can be captured by increasing the fraction of infused stem cells. Our simulations then suggest that the effects of increased fractions on resistance dynamics are qualitatively similar to those of proliferation enhancement, although they are more modest (see Figure S4). At any rate, a more elaborate modeling study of stem-cell delivered therapy is required to map the conditions that facilitate considerable responses. This exceeds the scope of this work but the ideas and models presented here are readily applicable to this case.

Gene therapy could also potentially be combined with HAART, so as to benefit from HAART-induced suppression. The problem with this approach is that HIV is the driving force behind the expansion of P cells, and their selective advantage over U cells becomes small with decreasing viral loads [42]. It thus appears that methods for stimulating P cell proliferation and/or selection must be developed, such that the P cells can accumulate independently of the infection, and can then be effectively combined with HAART. Moreover, these methods can then leverage on a broad range of agents, including a variety of RNA-based inhibitors (reviewed in [4]), which may not propel sufficient cell expansion, but do provide powerful viral suppression (see Text S1).

A number of simplifying assumptions were incorporated into our model to retain its generality and tractability. First, we assumed a fixed incremental effect of each subsequent mutation on a strain's phenotype. In reality, individual effects depend on the actual mutational composition and on order of appearance, and not all mutational routes yield a viable virus. We therefore neglected epistatic interactions between mutations and parameterized only the average incremental contribution. Second, we assumed that fitness in P cells is monotonically increasing with a mutant's order, and thus mutants of sufficiently high order always thrive under therapy conditions, and will likely emerge if generated. Other choices of fitness functions, such as ones that are non-monotonic or monotonic but assigning lower fitnesses to all strains, can render such high-order mutants less competent. Such choices would result in quantitatively different (more optimistic) emergence statistics, and would mostly depend on therapy effects on pre-existing mutants. Clearly, once data for specific therapies become available, the model should be revised to generate more precise predictions, but at this point, we focus on capturing the key features involved in the development of viral resistance, namely, the tradeoffs between the effects of 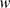 and 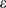. These qualitative findings do not depend on the monotonicity assumption since the same factors determine the ability of pre-existing strains to outgrow in the mixed-cell environment. The ideas and analysis pertaining to combinatorial approaches also apply regardless of the mutants order and are not function-specific. However, the effects of proliferation enhancement may differ under non-monotonic fitness assignments and should be revisited once relevant data are available.

We also assigned equal mutational fitness costs to replication in both cell types, which may not always apply. This implies that a strain is never fitter in P cells than in U cells, and matches recent experimental findings [23] (see the Methods section). However, if highly resistant strains replicate better in P cells, our predictions are over-optimistic - they provide upper bounds on therapy efficacy and overestimate 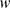
's impact on escape. Another simplification entails neglecting escape routes that are unlikely under HAART but may be selected in mixed cell compositions. Consider, for example, a strain with several mutations that partially resists therapy and is less fit than WT in U cells. Suppose that an additional mutation then partially rescues its fitness in U cells and possibly boosts its resistance as well. If such compensatory routes exist, they may be selected in environments where both fitness traits are valuable, and our assumption excluded such possibilities from consideration. Future work may further investigate the consequences of such complex fitness landscapes.

We extended our model to account for the contribution of recombination to the formation and loss of resistant strains. To maintain reasonable model complexity and size (especially for large 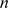
's), we included only simple recombination patterns, whereby mutations from distinct strains are adjoined, and mutations are lost during recombination with WT. The complete details are given in Text S1. We observed very little quantitative effects on the average treatment outcome, even under very high recombination rates. Therefore, in the interest of clarity and simplicity, we omitted recombination from the model. However, a more detailed treatment of recombination, particularly in conjunction with epistatic interactions, may reveal other outcomes, as it was previously shown that recombination effects are pronounced only in the presence of epistasis [76], [77]. A detailed analysis of this type requires a large dedicated model (see e.g. the work in [77]) and is not practical when studying a wide range of genetic barriers as was done here. Future work may incorporate such detailed mechanisms for special cases of interest. An additional extension we considered entailed a homeostasis control of thymus input rates as opposed to constant rates, but that did not qualitatively affect our results either (data not shown).

Many of our assumptions can be partially corroborated by *in vitro* assays. For example, it is common practice to characterize the spectrum of potentially resistant mutants through serial passage experiments. In our case, one can assess the attenuated infection rates from single-round infection assays [10], [44] and reconstruct an approximate “resistance profile”. Similar tests should be conducted with U cells to quantify both fitness traits. Assays with a mixed cell population can serve to probe the fitness landscape and identify mutants that trade their resistance to therapy for recovered fitness in U cells, as these may be selected *in vivo*. Experiments can also help determine the incremental contributions of single agents in combinatorial settings and whether they interact multiplicatively, additively, or in any other way. The real challenge is in extrapolating *in vitro* findings to clinical settings, since these do not fully reflect their *in vivo* counterparts, but yet are indicative of relative competence among strains. Clinical data are invaluable to validating our model, but to the best of our knowledge, sustained P cell expansion has not yet been reported in any clinical trial. Thus, experimental data from animal models [41], [78] can provide a starting point for better characterizing P cell expansion and viral suppression, by fitting variations of our model to clinical measurements.

To summarize, we use a generalized model of HIV gene therapy to identify both the most effective routes to control the virus and the critical properties of the antiviral genes that must be experimentally assessed during the design of such therapies.

## Methods

### Infection Dynamics

#### Baseline model

Our model extends upon a commonly used model of HIV dynamics [16], [17], which considers three entities: healthy target CD4+ T cells (*U*), productively infected T cells (*I_U_*), and free virions (*V*). We added two equations that track the densities of healthy P cells (*P*) and productively-infected P cells (*I_P_*). These follow similar dynamics, using modified parameter values. Additionally, we modified the T cell dynamics, such that two sources contribute to T cell renewal: a constant influx of naïve T cells from the thymus, and self-proliferation [37], [79], [80], [81]. Since we consider therapy delivery by T cell infusion, the thymus contribution is limited to U cells. The proliferation mechanism, in contrast, encompasses both cell types, and saturates according to Michaelis-Menten kinetics. This form was derived in [37] based on the idea that the activation and proliferation of T cells are confined by their competition for antigen-presenting sites. Our formulation also reflects an assumption that the *ex vivo* manipulation of P cells did not compromise their proliferative ability and that they display a normal T cell phenotype. Importantly, both cell types are assumed to equally contribute to saturation, thus equally competing for presence in the T cell pool. In the presence of gene therapy, the model dynamics are governed by the following equations:
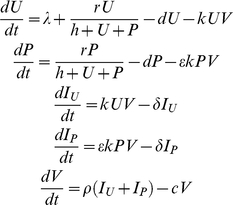
(2)where 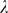 is the thymus-input rate, *r* and *h* are Michaelis-Menten constants, *d* is the lymphocyte death rate, *k* is the rate at which WT infects susceptible cells, 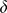 is the death rate of infected cells, *c* is the virion clearance rate, and 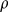 is the rate of virus production from infected cells. An additional parameter conveys the effects of therapy on P cells, that is, an inhibition factor 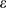, which attenuates the rate at which they are infected. If the proliferation of the P cells is enhanced, the rate is multiplied by an acceleration factor 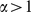, yielding a rate 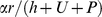. When stem cells are infused, the thymus input rate of U cells becomes 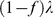, and a thymus input term of 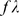 is added to the P cells' equation, where 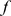 is the fraction of gene-modified stem cells.

The efficacy of early-stage inhibitory genes may be further enhanced by way of combination with late-stage inhibitory genes [12], [82]. When combined with a perfect viral production suppressor, the virus dynamics are governed by 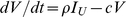. Steady-state analysis was carried out for this ideal case, which lends itself to simple analytical results. This model has three steady states: uninfected in the absence of therapy (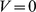, 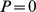), infected in the absence of therapy (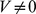, 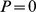), and infected in the presence of therapy (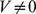, 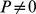). Since the viral load maintains the same ratio relative to the infected U cell density in all three cases (i.e., 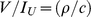), we compare the infected cell densities (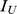) between the latter two states, instead of the viral loads. Specifically, we have 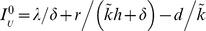 before therapy and 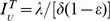 after therapy, where 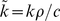 is a generalized infection rate combining viral infectivity, production, and clearance rates.

#### Suppression gain

It is clear from the above expression for 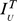 that the virus is not eradicated unless 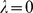. Otherwise, the viral suppression gain, or the ratio of cells infected in the absence vs. in the presence of therapy, is given by
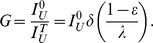
(3)Since 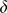 is well-characterized to be between 0.3 and 0.7 per day [38], [83] and the pre-therapy infected-cell levels are maximally on the order of a few tens per µL [84], the maximal suppression gain (when 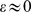) is approximately 10-fold greater than 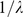. If we assume that 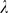 supplies no more than a few percent of the natural T-cell mortality in adults [45], this amounts to a contribution on the order of 0.01*0.01*1000 = 0.1 cells/[µL*day] (i.e., 1% of a death rate of 0.01/day multiplied by an average T-cell level of a healthy individual). Combining these estimates yields a maximal suppression gain of approximately two logs. To realize gains comparable to HAART [40], the thymus' relative contribution should be considerably lower than a few percent. We used Eq. (3) to illustrate the dependence of *G* on 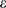 and 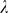 (Figure 2B–2C), while keeping the pre-therapy conditions fixed so as to allow for a fair comparison (see Text S1 for details).

At this point, we note that our choice of the baseline model was guided by a comparison we conducted between this model and a model of a different type of protection, whereby the genes inhibit expression of viral proteins in infected cells rather than infectivity. Such inhibition confers a selective advantage to the infected P cells in the form of extended life spans, and may additionally suppress virus production [20], [22]. Currently, a wide array of suitable methods exists, with some already being tested in clinical trials (reviewed in [4]). We found that for a wide range of relevant 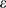 values, viral-entry inhibitors are more advantageous than gene-expression inhibitors (see Text S1 for details and for conditions under which the latter approach may be practical).

### Evolutionary Dynamics

#### Multistrain model

We extended the baseline model to account for the dynamics of a collection of viral strains, displaying a range of resistance capabilities to therapy. Three equations describe the dynamics of each mutant strain *M*, tracking the densities of infected U cells (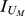), infected P cells (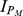), and free virions (*M*), as follows:
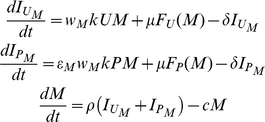
(4)where 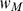 is a replicative fitness cost associated with mutating from WT, and 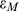 is the infectivity inhibition factor, expressing altered protection against strain *M*. The terms 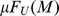 and 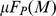 represent the mutation of other strains into *M* during reverse transcription, where 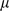 is the rate of point mutation at any resistance-conferring site, and 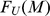, 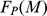 stand for the density of U and P cells that are newly infected with strains that may precede *M*, respectively. The infection terms 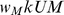 and 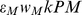 were subtracted from the susceptible-cell equations. Note that we assumed that viral production rates are unaffected by the mutational process, which is more likely to alter the fusion, binding, or integration capacities of the virus [10]. Our model formulation also neglects the loss of infected cells due to mutation into other strains. In preliminary simulations we observed that inclusion of such loss had little effect on our results.

#### Stochastic events

We supplemented Eq. (2) with a stochastic module that accounts for the randomness taking place during mutant appearance [18]. We followed previous approaches to implementing a stochastic counterpart of our model [85], [86], which takes effect whenever a species' density falls below a predefined threshold (similar to [86]). Additional details are found in Text S1.

#### Fitness cost

We modeled the replication fitness of a mutant as dependent on the number of resistance-conferring sites that are mutated away from their WT form. We refer to this number as the number of *resistance mutations*, or the *order* of the mutant. We assumed that fitness effects are multiplicative [53], implying a fitness cost of 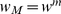 for an *m*-order mutant, where 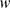 is a fixed average fitness cost per mutated site. We also assumed that this loss applies to infection of both U and P cells. This implies that for P cells, 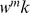 sets an upper limit on the infection rates of *m*-order mutants, as these may be further downgraded by a factor of 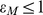. In other words, a strain cannot be fitter in P cells than in U cells. Similar relations, where mutants display weaker replication in P cells than in U cells, were recently observed *in vitro* for RNAi-escape mutants [23], and motivated our modeling choice. Nevertheless, this assumption may be optimistic, in the sense that sufficient adaptation may result in highly resistant strains that replicate better in P cells than in U cells. Our model can be readily adapted to reflect such conditions, but in the lack of relevant gene therapy data, we assumed that resistance manifests itself only through overcoming inhibition.

#### Infectivity inhibition

We assumed that a mutant's resistance level depends on the number of resistance mutations, with each additional mutation contributing equally and multiplicatively to overall resistance. We therefore spaced the inhibition factors of intermediate mutants between the WT and full-resistance levels, as follows. If *n* mutations confer full resistance, then the inhibition factor of an *m*-order mutant (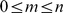) is set to 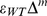, where 
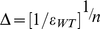
, and 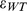 stands for WT inhibition. Therefore, the fully-resistant *n*-order mutant experiences no inhibition by P cells, and is equally competent in both cell types. Note that such scheme disregards epistatic effects and is equivalent to a linear change in viral infectivity on a logarithmic scale.

Almost no quantifiable data is available on the efficacy of gene therapies against increasingly resistant mutants, making it difficult to accurately model the changes in 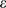. However, the reasoning behind our choice is twofold. First, if several protective genes are combined, we assume their inhibitory effects are multiplicative. Partial resistance can then be thought of as escaping a subset of therapies [86]. Second, recent experiments with traditional antiretroviral therapies support a similar multiplicative form of declining efficacy with mutation accumulation [54], [77], [87]. In the context of gene therapy, we are aware of one recent work that partially quantified the infectivity inhibition of intermediate mutants under *in vitro* conditions [10], indicating that single-mutation contributions are non-additive and nonlinear, thus further supporting our modeling assumption. Moreover, our understanding of the mechanism of HIV's escape from RNA-mediated inhibition [11], [22], where potency correlates with the goodness of match between the genome and the target sequence, further motivates our choice.

#### Mutations

Our model accounts only for first-order accrual of point mutations, that is, each strain may be added at most a single point mutation at each ODE integration step (Figure 1B). For simplicity, we also excluded the reverse mutational process (i.e., towards less-resistant strains), as its contribution is minor due to much-lower densities of higher-order mutants. Our assumptions thus imply that an *m*-order mutant may be directly preceded by each of the (*m*−1)-order mutants whose mutated sites form a subset of its *m* sites. For example, if 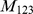 is 3-point mutations away from WT (the subscripts label the mutated sites), then its predecessors are the 2-order mutants 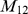, 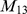, and 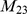.

#### Complexity reduction

The above assumptions allude to perfect symmetry among same-order mutants, as any *m*-order mutant experiences a fitness cost factor of 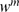 and an inhibition factor of 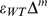. We exploited it to obtain a smaller and simplified model. For each *m*, we grouped all *m*-order strains into one class and summed the various densities over all class mutants. The resulting equations appear as follows:
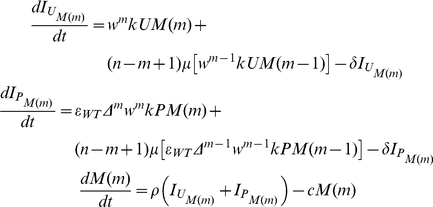
(5)where *n* is the order of the fully-resistant mutant, and 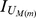, 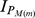, and 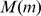 are the aggregate densities per *m*. The multiplicity coefficient (*n*−*m*+1) accounts for the affiliation of each (*m*−1)-order strain with the predecessor sets of multiple *m*-order strains. For example, when *n* = 3, the mutant 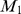 contributes to the influx of both 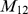 and 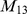 (*m* = 2), whereas WT contributes to 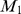, 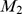, and 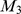 (*m* = 1). The symmetric model has 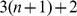 equations, compared to 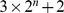 in the general case. Such complexity reduction becomes significant when considering the large *n*'s required for effective gene therapy, and is crucial for tackling the more elaborated scenario of multiple P-cell populations.

### Heterogeneous Protected-Cell Composition

We assumed the availability of two distinct protective-gene cassettes, which target different viral components, and thereby give rise to two non-overlapping sets of resistance-conferring sites, 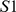 and 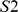. We further assumed that both cassettes, hereafter called Therapy 1 and Therapy 2, are equally powerful, that is, they have the same starting inhibition factor 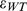, same *n*, and same intermediate inhibition factors. When used jointly, there is no cross-resistance between them. That means that the inhibition exerted by Therapy 1 (2) on a given strain depends solely on the number of mutated sites in 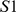 (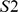), and is independent of the mutated sites in 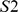 (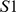). For a given *n*, there are now 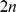 resistance-conferring sites, contained in 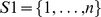 and 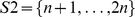. A strain that shares *m* mutated sites with 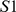 and *l* mutated sites with 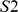 will encounter inhibition factors of 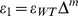 and 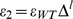 by Therapies 1 and 2, respectively. The fitness cost factor is determined by the total number of mutated sites, and equals 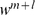.

#### Symmetric form

A straightforward implementation of such model encompasses 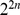 strains and 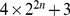 equations. As before, we exploited the inherent symmetry and classified the strains into 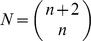 resistance-phenotype classes. In particular, each class is identified by a pair 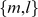 (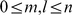), and all its members share *m* sites with one therapy's set and *l* sites with the other set (see Text S1 for details and equations). The model has 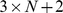 equations (which is on the order of 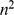) and takes a similar form to Eq. (4), with modified inhibition and fitness cost factors, predecessor classes, and multiplicity coefficients. The major reduction in model size ensured the tractability of the model and the simulations for relevant *n* values.

### Model Parameters and Initialization

We used the following parameter values based on prior studies [70], [83], [84], [86], [88], [89], [90]: the death rate of healthy CD4+ cells, 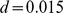 1/day; the death rate of infected cells, 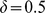 1/day; the viral clearance rate, 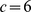 1/day; the mutation rate, 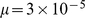 per base per replication. The virion production and infection rates are not well characterized and were set to 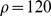 virions/[cell*day] and 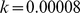 µL/[virion*day], respectively, to match observed viral dynamics in untreated infections. We set the T-cell renewal parameters to yield realistic T cell densities in the absence of infection (1,000–1,300 cells/µL) and realistic pre-therapy viral loads (10^2^–10^3^ virions/µL) [16], [38], [90]. We also assumed that the thymus regenerates 1% of the total cell loss, implying the following parameter values: 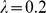 cells/[µL*day], 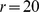 cells/[µL*day], and 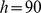 cells/µL. We also tested numerous other parameter values and did not observe qualitatively different results. A species was considered extinct if its density fell below 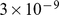 per µL, corresponding to one entity per body, based on 5 liters of blood which contain 1%–2% of the entire susceptible cell population [86]. Its dynamics were treated stochastically as long as its density did not exceed 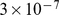 per µL. The default values of therapy-related parameters were 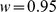 and 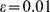 [44]. The fraction of infused P cells was 1% of the entire U cell population at the time of infusion (day 0) [5], [21]. The system was first brought to steady state, where simulations over a 400-day period ensured convergence to a HIV set point. Our parameter choices result in pre-existing strains with one, two, and three resistance mutations. Clinical experience suggests that double-point mutations are likely to exist in sufficient levels in untreated HIV patients, but it is not known if triple-point mutations are sufficiently abundant as well. Our model may thus be too “pessimistic”, and under less conservative parameter choices will result in less stringent requirements for a successful gene therapy.

## Supporting Information

Figure S1 Stochastic effects that dominate mutant appearance result in large variations in fixation times. Effects of increasingly larger genetic barriers n (x-axis) on the time to fixation (y-axis), which is the time required for the resistant strains to reach 50% of the viral population, measured from therapy initiation at day 0. Each marked point represents the average of at least 50 simulation runs in which fixation occurred within four years from therapy initiation. The bars correspond to one standard deviation. Each curve depicts the aggregate outcome for different parameter choices, where w is a fixed average fitness cost per mutated site and ε is the therapy's inhibition factor (see the Methods section for more details). All other parameters were set to their default values. The figure shows that larger n's are associated with increasing variation in fixation time. This is because the stochastic fluctuations of newly emerging mutants build up along the evolutionary process. When n becomes larger, more mutations are needed in order to confer sufficient resistance, and hence the time to fixation of such highly-mutated strains depends on the appearance times of their preceding mutants, which experience random drift as well.
(0.05 MB PDF)

Figure S2 Infection dynamics under enhanced proliferation of protected cells. System dynamics in the absence of viral resistance and when the P cells have a proliferative advantage over the U cells. Enhanced proliferation of P cells was modeled as a constant percentage increase in their proliferation rate compared to the U cells' proliferation rate. Simulations were performed with default parameter values and with 30% increase in the P cells' proliferation rate (see the Methods section for more details). Enhanced proliferation expedites P cell accumulation and viral decline and drives the virus to extinction. It additionally results in higher P cell densities, allowing them to increase their relative proportion in the immunity system on the expense of a declining U cell population.
(0.16 MB PDF)

Figure S3 Infection dynamics following infusion of a large fraction of protected stem cells. System dynamics in the absence of viral resistance and when protected stem cells are infused to the patient. Stem cell infusion was modeled by an addition of a constant thymus-export term to the renewal of P cells, and by a reduction in the rate of thymus export to the U cell compartment. The modified terms are determined by the fraction of the protected stem cells in the bone marrow (see the Methods section). The simulation corresponds to 90% of the stem cells being protected, and to a bone-marrow contribution of 10%, and was performed with default infection and therapy parameter values. Increasing the fraction of protected stem cells expedites P cell accumulation and viral decline, and allows for higher P cell densities to be reached, such that their relative proportion in the immunity system is increased on the expense of the declining U cell population.
(0.06 MB PDF)

Figure S4 Effects of increasing the fraction of protected stem cells. Success rates (A), average fixation times (B), and average viral reductions (C) are shown as functions of the genetic barrier n (x-axis) for a range of infused fractions. The parameter f corresponds to the fraction of stem cells that are protected (see the Methods section), and different colors depict different f values. Each point summarizes the outcomes of 500 simulation runs with default parameter values. Success rates gradually improve with increasing protected fractions (A), but when the virus escapes, fixation is generally expedited (B). The viral reductions obtained by the time of fixation are also improved (C).
(0.03 MB PDF)

Text S1 Additional details about the model and simulations.
(0.43 MB DOC)
