# Supplementary figures and images for: Computational Models of HIV-1 Resistance to Gene Therapy Elucidate Therapy Design Principles
Source: PLoS Comput Biol. 2010 Aug 12;6(8):e1000883. doi: 10.1371/journal.pcbi.1000883 (PMC2920833; doi:10.1371/journal.pcbi.1000883)

Average time (days)

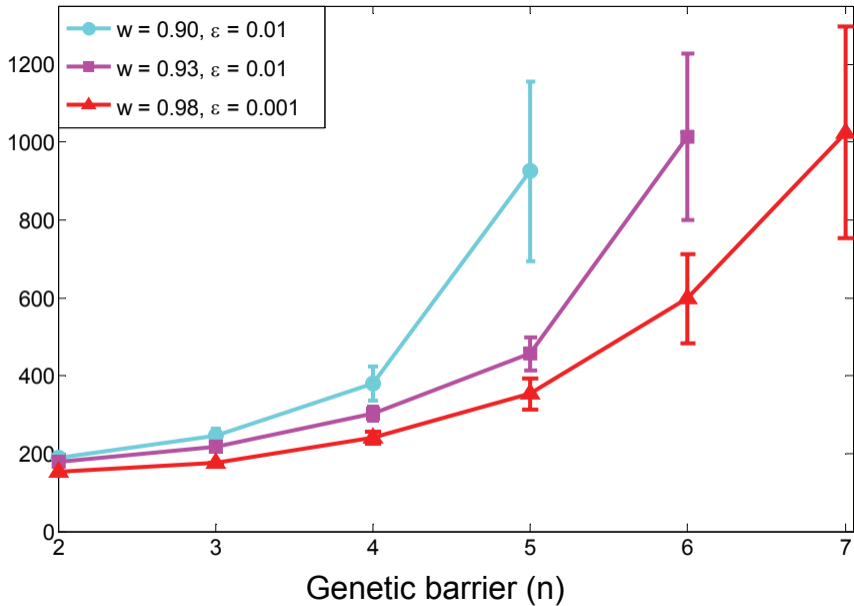

Supplement: Figure S1 — Stochastic effects that dominate mutant appearance result in large variations in fixation times. Effects of increasingly larger genetic barriers n (x-axis) on the time to fixation (y-axis), which is the time required for the resistant strains to reach 50% of the viral population, measured from therapy initiation at day 0. Each marked point represents the average of at least 50 simulation runs in which fixation occurred within four years from therapy initiation. The bars correspond to one standard deviation. Each curve depicts the aggregate outcome for different parameter choices, where w is a fixed average fitness cost per mutated site and ε is the therapy's inhibition factor (see the Methods section for more details). All other parameters were set to their default values. The figure shows that larger n's are associated with increasing variation in fixation time. This is because the stochastic fluctuations of newly emerging mutants build up along the evolutionary process. When n becomes larger, more mutations are needed in order to confer sufficient resistance, and hence the time to fixation of such highly-mutated strains depends on the appearance times of their preceding mutants, which experience random drift as well. (0.05 MB PDF) [file pcbi.1000883.s001.pdf]

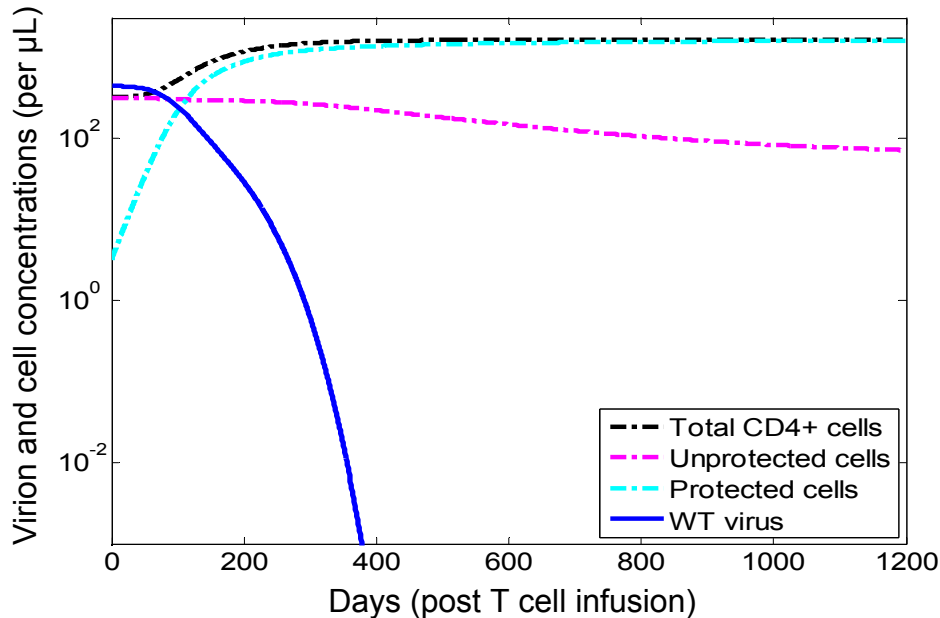

Supplement: Figure S2 — Infection dynamics under enhanced proliferation of protected cells. System dynamics in the absence of viral resistance and when the P cells have a proliferative advantage over the U cells. Enhanced proliferation of P cells was modeled as a constant percentage increase in their proliferation rate compared to the U cells' proliferation rate. Simulations were performed with default parameter values and with 30% increase in the P cells' proliferation rate (see the Methods section for more details). Enhanced proliferation expedites P cell accumulation and viral decline and drives the virus to extinction. It additionally results in higher P cell densities, allowing them to increase their relative proportion in the immunity system on the expense of a declining U cell population. (0.16 MB PDF) [file pcbi.1000883.s002.pdf]

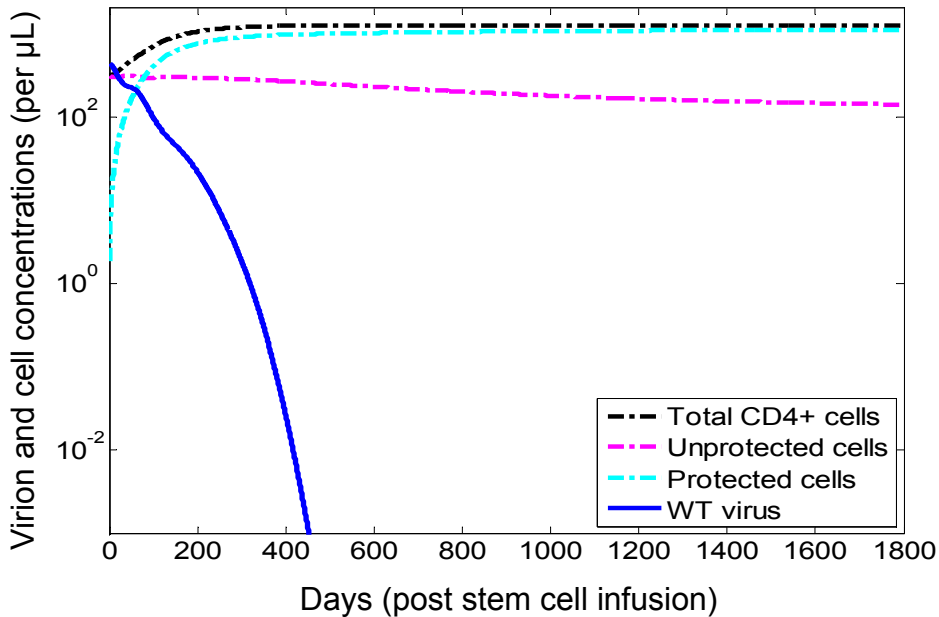

Supplement: Figure S3 — Infection dynamics following infusion of a large fraction of protected stem cells. System dynamics in the absence of viral resistance and when protected stem cells are infused to the patient. Stem cell infusion was modeled by an addition of a constant thymus-export term to the renewal of P cells, and by a reduction in the rate of thymus export to the U cell compartment. The modified terms are determined by the fraction of the protected stem cells in the bone marrow (see the Methods section). The simulation corresponds to 90% of the stem cells being protected, and to a bone-marrow contribution of 10%, and was performed with default infection and therapy parameter values. Increasing the fraction of protected stem cells expedites P cell accumulation and viral decline, and allows for higher P cell densities to be reached, such that their relative proportion in the immunity system is increased on the expense of the declining U cell population. (0.06 MB PDF) [file pcbi.1000883.s003.pdf]

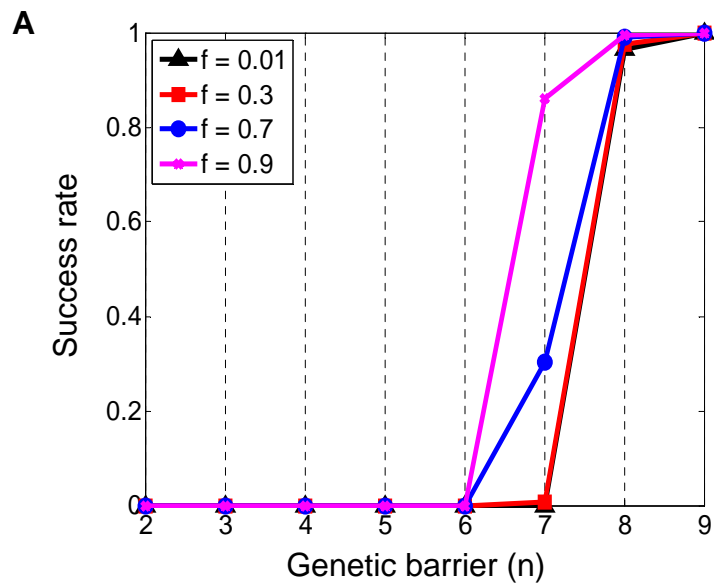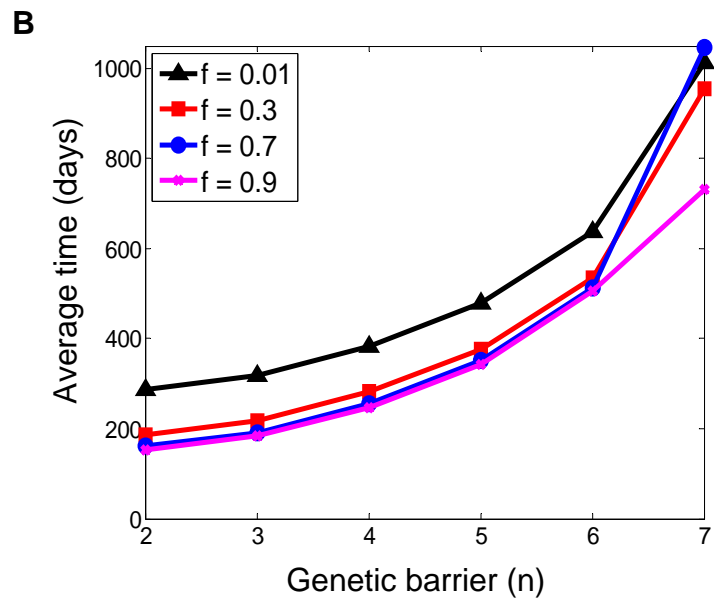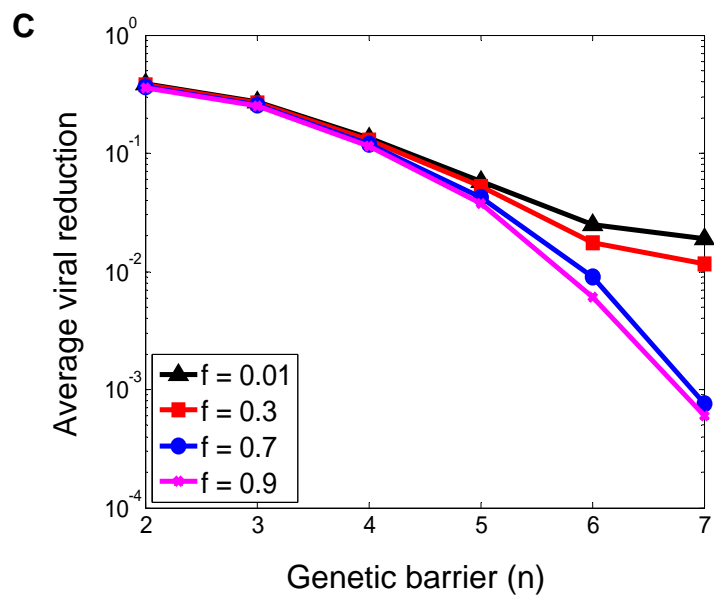

Supplement: Figure S4 — Effects of increasing the fraction of protected stem cells. Success rates (A), average fixation times (B), and average viral reductions (C) are shown as functions of the genetic barrier n (x-axis) for a range of infused fractions. The parameter f corresponds to the fraction of stem cells that are protected (see the Methods section), and different colors depict different f values. Each point summarizes the outcomes of 500 simulation runs with default parameter values. Success rates gradually improve with increasing protected fractions (A), but when the virus escapes, fixation is generally expedited (B). The viral reductions obtained by the time of fixation are also improved (C). (0.03 MB PDF) [file pcbi.1000883.s004.pdf]
