# Supplementary material for: Computational Models of HIV-1 Resistance to Gene Therapy Elucidate Therapy Design Principles
Source: PLoS Comput Biol. 2010 Aug 12;6(8):e1000883. doi: 10.1371/journal.pcbi.1000883 (PMC2920833; doi:10.1371/journal.pcbi.1000883)
Supplement: Text S1 — Additional details about the model and simulations. (0.43 MB DOC) [file pcbi.1000883.s005.doc]

**Text S1**: Additional Details about the Model and Simulations

Hybrid Model Implementation

We describe HIV-1’s evolutionary dynamics with a hybrid stochastic-deterministic model similar to the one used by Curlin *et al*. [1]. The idea is to determine the trajectories of large populations deterministically while treating small populations stochastically. A pre-defined population-size threshold is used to determine which integration method is to be used for advancing the trajectory of any given species. An additional threshold (called an extinction threshold) corresponds to a density of a single entity in the system, and is used to determine when to remove a species. Integration is performed in fixed and very small time steps. A species whose density is larger than the population-size threshold is integrated deterministically according to $x(t+h)=x(t)+\frac{dx}{dt}(t)h$, where $h$ is the step size, $x(t)$ is its density at time $t$, and $\frac{dx}{dt}(t)$ corresponds to its differential-equation description in the model. For each species at densities below the population-size threshold, we list the changes that may occur to its population in a sufficiently small time interval. These include the birth and death of a single entity, and the lack of change to the population. Each event is assigned a probability, based on the rates in the deterministic equation $\frac{dx}{dt}$, as follows. The probability of a birth or death event equals $r^{x}(t)h$, where $r^{x}(t)$ is the deterministic birth or death rate, respectively. The probability that none of the former events will occur equals 1 minus the sum of the birth and death probabilities. If the calculation does not result in a probability distribution, the step size $h$ is made smaller until a proper distribution is generated. A draw from this distribution determines the state of the species at time $t+h$. In the simulations reported here, $h$ did not exceed an interval of two seconds and was occasionally made smaller.

Modeling Recombination

We modeled recombination by adding selected dual infections and heterozygote virions to the symmetric model. Recombination was expressed by adding or subtracting terms of heterozygote infections from the equations that track cells infected with *n*-order strains. These terms were multiplied by a constant, reflecting the probability that a heterozygote will go through a sequence of recombination events that results in exactly *n* mutations.

Specifically, for a genetic barrier *n*, we added $3\times\left(\left\lfloor n/2\right\rfloor+1\right)$ ($\left\lfloor x\right\rfloor$ stands for floor of $x$) states to the symmetric model. The states correspond to cells (*U* and *P*) that are dually infected by virions from classes *j* and $n-j$, where $0\leq j\leq\left\lfloor n/2\right\rfloor$, and to the heterozygous virions budding out of them. Each such additional “dual class” is denoted by $D\left(j,n-j\right)$. Dual infections were incorporated by adding the following equations:

|  | $\begin{matrix} \frac{dI_{U_{_{M\left(D\left(j,n-j\right)\right)}}}}{dt}=0.7\left[\begin{matrix} w^{j}kM(j)I_{U_{M(n-j)}} \\ +w^{n-j}kM(n-j)I_{U_{M(j)}} \end{matrix}\right]-\delta I_{U_{_{M\left(D\left(j,n-j\right)\right)}}} \\ \frac{dI_{P_{_{M\left(D\left(j,n-j\right)\right)}}}}{dt}=0.7\varepsilon_{WT}\left[\begin{matrix} \Delta^{j}w^{j}kM(j)I_{P_{M(n-j)}} \\ +\Delta^{n-j}w^{n-j}kM(n-j)I_{P_{M(j)}} \end{matrix}\right]-\delta I_{P_{_{M\left(D\left(j,n-j\right)\right)}}} \end{matrix}$ | (6) |
| --- | --- | --- |

where a dual infection may be obtained by *j*-order virions infecting cells already infected with ($n-j$)-order virions or vice versa. A coefficient of 0.7 accounts for downregulation of the CD4 receptor following infection of a susceptible cell [2]. Production of heterozygotes follows the same dynamics as before, with half of the production rate to account for the fact that approximately half of the packaged virions are heterozygous [3].

The heterozygous virions may infect susceptible U and P cells, a step that may eventually lead to a recombinant form with *n* mutated sites. However, only some occurrences of recombination events at specific locations would result in the desired *n*-order mutant, and hence only a fraction of the successful infections in fact yields an *n*-order mutant. We accommodated such a probabilistic event by multiplying the infection terms by a constant factor denoted *recomb* ($recomb\leq1$), representing the likelihood of generating a “correct” recombinant form. The following infection terms were then added to the equations describing cells that are infected with *n*-order mutants ($I_{U_{M(n)}}$ and $I_{P_{M(n)}}$):

$$recomb\times\frac{(w^{j}+w^{n-j})}{2}kUM\left(D\left(j,n-j\right)\right)$$

and

$recomb\times\varepsilon_{WT}\Delta^{n-j}\frac{(w^{j}+w^{n-j})}{2}kPM\left(D\left(j,n-j\right)\right)$,

respectively, for each *j*, $1\leq j\leq\left\lfloor n/2\right\rfloor$. These terms represent the most likely paths to generation of *n*-order strains, that is, via recombination of lower-order strains.

Here, the fitness loss was taken as the average between the two genomes, and the inhibition was set to be equal to the inhibition experienced by the more-resistant strain ($n-j$) (thus representing a worst-case scenario). The infection terms for $1\leq j\leq\left\lfloor n/2\right\rfloor$ constitute an additional source of *n*-order mutants. In the special case when $j=0$, the corresponding infection term was subtracted from both equations (for $I_{U_{M(n)}}$ and $I_{P_{M(n)}}$), as recombination with WT may lead to a decreased-order mutant. Since without further assumptions it is difficult to determine the probability of encountering all feasible recombination events, we simulated the two extreme cases with probabilities (*recomb*) zero and one.

Note that to retain manageable model sizes for relevant *n*’s, we modified the symmetric model in an iterative manner. We first modified the model for $n=2$, and consequently observed that recombination effects were quantitatively negligible. We subsequently modified the model for $n=3$ to account for recombination into 3-order mutants, while neglecting recombination into 2-order mutants. We followed a similar procedure for $n=4$. In all cases, perfect recombination ($recomb=1$) resulted in a slight increase in average fixation times for pre-exiting mutants ($n\leq3$), and a slight decrease for non-existing mutants, both of about one day out of several hundreds days (data not shown). This suggests that the contribution of recombination is insignificant within the context of our model, and due to the largely increased complexity of such extensions, we excluded recombination from our model and all other simulations.

Model of a Heterogeneous Protected-Cell Composition

As explained in the main text, Therapies 1 and 2 are identical, entailing the same genetic barrier *n* and obeying the same inhibition factor scheme. If a mutant has *m* mutated sites in set $S1=\left\{1,\ldots,n\right\}$ and *l* mutated sites in set $S2=\left\{n+1,\ldots,2n\right\}$, then it experiences inhibition factors of $\varepsilon_{1}=\varepsilon_{WT}\Delta^{m}$ and $\varepsilon_{2}=\varepsilon_{WT}\Delta^{l}$ by Therapies 1 and 2, respectively. Its fitness loss is determined by the total number of mutated sites and equals$w^{m+l}$.

Since inhibition and fitness loss are determined solely by the number of mutated sites that a mutant shares with $S1$ and $S2$, and since both therapies have the same *n*, there is an additional perfect symmetry between the two types of P cells, $P1$ and $P2$. Specifically, the replication of a mutant with $m$ positions in $S1$ and $l$ positions in $S2$ over $P1$ cells is identical to the replication of a mutant with $m$ positions in $S2$ and $l$ positions in $S1$ over $P2$ cells. Consequently, the density of $P1$ cells infected with all mutants with $m$ positions in $S1$ and $l$ positions in $S2$ (i.e., class $M\left(\left(m,l\right)\right)$) equals the density of $P2$ cells infected with all mutants with $m$ positions in $S2$ and $l$ positions in $S1$ (i.e., class $M\left(\left(l,m\right)\right)$). This suggests that both quantities are indistinguishable, and can be lumped together into an “overall density of protected cells, infected with all mutants possessing *m* sites in one therapy’s set and *l* sites in the other therapy’s set”. In other words, there is no importance to the order of *m* and *l*’s assignments. We can thus group the mutants according to this criterion, and consider the total density of P cells ($P1$ and $P2$) that are infected by each class, with no distinction between the protection identities. We identify each viral class by a pair$\left\{m,l\right\}$ and denote the aggregate density of its member strains by $M\left(\left\{m,l\right\}\right)$.

The replication dynamics of class $\left\{m,l\right\}$ over U cells can be described by

|  | $\begin{matrix} \frac{dI_{U_{_{M\left(\left\{m,l\right\}\right)}}}}{dt}=w^{m+l}kUM\left(\left\{m,l\right\}\right) \\ \text{ }+\mu w^{m+l-1}kU\left[\begin{matrix} A_{M\left(\left\{m-1,l\right\}\right)}M\left(\left\{m-1,l\right\}\right) \\ +A_{M\left(\left\{m,l-1\right\}\right)}M\left(\left\{m,l-1\right\}\right) \end{matrix}\right]-\delta I_{U_{_{M\left(\left\{m,l\right\}\right)}}} \end{matrix}$ | (7) |
| --- | --- | --- |

where the multiplicity coefficients are given by

|  | $A_{M\left(\left\{m-1,l\right\}\right)}=\left\{\begin{matrix} 2(n-m+1) & \text{ if }m-1=l \\ n-m+1 & \text{otherwise} \end{matrix}\right.$ $A_{M\left(\left\{m,l-1\right\}\right)}=\left\{\begin{matrix} 2(n-l+1) & \text{ if }m=l-1 \\ n-l+1 & \text{otherwise} \end{matrix}\right.$. | (8) |
| --- | --- | --- |

Sequential accumulation of mutations dictates that members of class $\left\{m,l\right\}$ arise from infections by members of classes $\left\{m-1,l\right\}$ and $\left\{m,l-1\right\}$, as long as $m-1,\text{ }l-1\geq0$. Since each member of any preceding class may evolve into several $\left\{m,l\right\}$ mutants (by altering any of its non-mutated sites), its contribution appears multiple times when summing over all $\left\{m,l\right\}$ mutants. Consider, for example, the case when the genetic barrier is $n=2$, and consider the $\left\{0,2\right\}$ mutant $M_{12}$ (i.e., only $S1=\{1,2\}$ sites are mutated, while the $S2=\{3,4\}$ sites assume their WT form). This mutant may evolve into two mutants: $M_{123}$ or $M_{124}$, depending on which site in $S2=\{3,4\}$ is mutated. Class $\left\{0,2\right\}$ should therefore be counted twice in the mutation-accumulation term of class $\left\{1,2\right\}$. In general, there are $n-(m-1)$ possibilities to extend an $\left\{m-1,l\right\}$ mutant into $\left\{m,l\right\}$ mutants, as this is the number of non-mutated sites that one can choose from. The only exception is when $m-1=l$. In this case, consider the $\left\{1,1\right\}$ mutant $M_{13}$, which may turn into$M_{123}$ or $M_{134}$ (both are $\left\{1,2\right\}$-members), depending on the set in which the acquired mutation is located. Thus, this class should also be counted twice in the mutation-accumulation term of class $\left\{1,2\right\}$. In general, the added site can be any of the non-mutated sites in *both* sets, implying $2\left(n-(m-1)\right)$ possibilities for extension into $\left\{m,l\right\}$ mutants. Similar logic applies to mutating $\left\{m,l-1\right\}$ members into $\left\{m,l\right\}$ members.

The dynamics of replication over P cells follow similar lines:

|  | $\begin{matrix} \frac{dI_{P_{_{M\left(\left\{m,l\right\}\right)}}}}{dt}=\frac{\varepsilon_{WT}(\Delta^{m}+\Delta^{l})}{2}w^{m+l}kPM\left(\left\{m,l\right\}\right) \\ \text{ }+\mu w^{m+l-1}kP\left[\begin{matrix} A_{M\left(\left\{m-1,l\right\}\right)}\frac{\varepsilon_{WT}(\Delta^{m-1}+\Delta^{l})}{2}M\left(\left\{m-1,l\right\}\right) \\ +A_{M\left(\left\{m,l-1\right\}\right)}\frac{\varepsilon_{WT}(\Delta^{m}+\Delta^{l-1})}{2}M\left(\left\{m,l-1\right\}\right) \end{matrix}\right] \\ \text{ }-\delta I_{P_{_{M\left(\left\{m,l\right\}\right)}}} \end{matrix}$ | (9) |
| --- | --- | --- |

where now the inhibition factors are also integrated into the infection terms. Since we combined both protected cell types into one population, their corresponding inhibition factors were averaged to yield a composite factor, as appears in the equation above. The mathematical description of free virion densities per each class is a straightforward extension of the homogeneous population case, as follows:

|  | $\frac{dM\left(\left\{m,l\right\}\right)}{dt}=\rho\left(I_{U_{_{M\left(\left\{m,l\right\}\right)}}}+I_{P_{_{M\left(\left\{m,l\right\}\right)}}}\right)-cM\left\{m,l\right\}$. | (10) |
| --- | --- | --- |

The symmetric model entails a compact simplification of a complex quasispecies environment, and limits consideration to $\left(\begin{matrix} n+2 \\ n \end{matrix}\right)$ viral classes and integration to $3\times\left(\begin{matrix} n+2 \\ n \end{matrix}\right)+2$ variables. In general, if one utilizes *K* distinct but equally powerful therapies, the viral strains can be classified into $\left(\begin{matrix} n+K \\ n \end{matrix}\right)$ classes, which differ in their resistance phenotypes. As explained above, each class corresponds to an unordered set of *K* integers, representing the numbers of mutated sites per each of the *K* therapies, but with no specific assignment of numbers to therapies. Each of the *K* numbers can range from 0 to *n*, where at the extremes we have WT with all zeros and the fully-resistant mutant with all *n*’s. The number of classes equals the number of *K*-sets with elements taken from a set of cardinality *n*+1, that is $\left(\begin{matrix} n+K \\ n \end{matrix}\right)$. Finally, we note that one can easily extend the above procedure to any number *K* of protected cell sub-populations, with minor modifications to the multiplicity coefficients and to the averaged inhibition factors.

The model simulations were performed with a code written in C that is available upon request from the authors.

Replication Fitness over a Heterogeneous Target Environment

Following previous works [4,5], we simplify the model in Eq. (2) in the main text by excluding the virus from it. This simplification is based on empirical evidence that the virus dynamics are typically fast in comparison to those of the infected cells [6]. Since the virus responds quickly to changes in the infected cell level, it is considered to be at quasi-steady state with respect to the infected cells. Such local equilibrium implies that the virus density is proportional to the corresponding infected-cell densities, as follows: $V=\frac{\rho}{c}\left(I_{U}+I_{P}\right)$. If we plug this identity into Eq. (2) and collect terms, our baseline model reduces to the following equations:

|  | $\begin{matrix} \frac{d(U+P)}{dt}=\lambda+\frac{r(U+P)}{h+U+P}-d(U+P)-\tilde{k}(I_{U}+I_{P})(U+\varepsilon P) \\ \frac{d(I_{U}+I_{P})}{dt}=\tilde{k}(I_{U}+I_{P})(U+\varepsilon P)-\delta(I_{U}+I_{P})=\left[\tilde{k}(U+\varepsilon P)-\delta\right](I_{U}+I_{P}) \end{matrix}$ | (11) |
| --- | --- | --- |

where $\tilde{k}=k\rho/c$ is a generalized infection rate combining viral infectivity, production, and clearance rates, and all other parameters are the same as in the main text.

The mathematical relation $\frac{d(I_{U}+I_{P})}{dt}=\left[\tilde{k}(U+\varepsilon P)-\delta\right](I_{U}+I_{P})$ describes the growth of the virus, which is generated by both infected cell types. It provides a measure of the virus replication fitness within the modeled environment. If we further assume that $U$ and $P$ remain fixed throughout therapy, then this relation can be extrapolated into an exponential growth function with a rate $\tilde{k}(U+\varepsilon P)-\delta$. Note that after P cell expansion (which takes approximately 200 days), $U$ and $P$ are indeed nearly constant. When *P* and/or *U* are not fixed, then one can think of the viral growth as behaving “instantaneously” exponentially, with a growth rate that changes over time. We also wish to stress that *P* is in fact $P(\varepsilon)$, as more potent therapies reach higher P cell densities and indirectly affect the fitness in this way. However, $P(\varepsilon)$ changes very little within the range of $\varepsilon$ we consider here, and in the interest of simplicity, we omitted this dependence from the equation.

The same idea applies to our multistrain model, since the density of any viral strain $M$ is proportional to the total density of the cells infected with it (i.e., $M=\frac{\rho}{c}\left(I_{U_{M}}+I_{P_{M}}\right)$). If we further neglect the generation of a strain from other strains by mutation (that is, the terms $\mu F_{U}(M)$and $\mu F_{P}(M)$ in Eq. (4) in the main text), then the growth of a strain $M$ is described by

|  | $\frac{d\left(I_{U_{M}}+I_{P_{M}}\right)}{dt}=\left[w_{M}\tilde{k}(U+\varepsilon_{M}P)-\delta\right]\left(I_{U_{M}}+I_{P_{M}}\right)$ | (12) |
| --- | --- | --- |

where $w_{M}$ and $\varepsilon_{M}$ are the replicative fitness cost and the infectivity-attenuation factor that apply to strain $M$, respectively. The replication fitness that is presented in Eq. (1) in the main text is the instantaneous growth rate of strain $M$, as derived from Eq. (12).

Finally, we wish to point out a subtle difference between our use of the replication fitness notion and its prior use in interpreting resistance to traditional drugs. This concept was originally developed as a tool for quantifying the relative fitness between strains based on *in vitro* measurements. It was derived from models of viral competition assays, which are initialized with equal amounts of virions for each competing strain. In this case, the mutational influx from one strain to another is negligible, and is therefore ignored. Although we followed this approach and neglected the mutational influx terms which contribute to a strain’s growth, in our case, this constitutes a cruder approximation due to a higher relative abundance of the predecessor strains. Despite the fact that this influx amounts to a small fraction of what is generated by new infections (it is on the order of several percent for pre-existing strains), it can make a non-negligible difference to the overall fitness expression (that is, after subtracting $\delta$). However, the extent of approximation is fairly similar for the various strains, and as we demonstrated in the main text, this concept still provides a powerful tool for understanding viral dynamics *in vivo*.

Model of Gene Therapy Targeted at Viral Gene Expression

Genes that inhibit expression of viral proteins take effect after viral integration, and hence act within cells that are already infected. Interfering with HIV replication in this manner can have two outcomes: reducing its effective progeny size and/or reducing its adverse effects on the host cell such as cell cycle arrest and cell death by necrosis or apoptosis [7]. While the first outcome serves to suppress viral replication, the latter one recovers the infected cells’ normal functionality and facilitates their expansion *in vivo*. As explained in the main text, such expansion is essential in order to reach meaningful fractions of protected cells, and is mediated by a selective advantage the infected cells can have over their healthy counterparts. Here, such advantage stems from the fact that the infected cells are constantly generated throughout the infection at the expense of the healthy cells that are lost. If their life spans and proliferation rates do not differ significantly from those of the healthy cells, the infected P cells can potentially increase their relative abundance in the immune system.

To isolate the effects of alleviating adverse viral effects and to explore the potential limits of this type of inhibition, we considered an “ideal” therapy in which virion production is fully suppressed in infected P cells. Therapy effects are expressed by decreased death rates and by recovered proliferation ability of the infected P cells. An inhibition factor $\varepsilon$ determines the extent of these improvements. The following equations summarize the dynamics under such treatment:

|  | $\begin{matrix} \frac{dU}{dt}=\lambda+\frac{rU}{h+U+P+I_{U}+I_{P}}-dU-kUV \\ \frac{dP}{dt}=\frac{rP}{h+U+P+I_{U}+I_{P}}-dP-kPV \\ \frac{dI_{U}}{dt}=kUV-\delta I_{U} \\ \frac{dI_{P}}{dt}=kPV+(1-\varepsilon)\frac{rI_{P}}{h+U+P+I_{U}+I_{P}}-\left[d+\varepsilon(\delta-d)\right]I_{P} \\ \frac{dV}{dt}=\rho I_{U}-cV \end{matrix}$ | (13) |
| --- | --- | --- |

where all the parameters are the same as in the main text. Note that the homeostatic mechanism now incorporates also infected cells ($I_{U}$ and $I_{P}$), since the infected P cells may occupy significant portions of the immune system and should be accounted for. The infected cells were not included previously since in all investigated scenarios they amounted to negligible fractions of the healthy cells, and thus their inclusion in the homeostatic mechanism was insignificant. For the purpose of fair comparison with the early-stage inhibition model, we also incorporated the same proliferation function into the model in Eq. (2) in the main text. Such modification does *not* alter the post-therapy viral load and results in a slight decrease in the pre-therapy load.

In the presence of therapy, the unprotected infected cells’ density at steady state equals $I_{_{U}}^{T}=\frac{\lambda}{\delta}+\frac{\delta}{\tilde{k}}\times\frac{\varepsilon}{1-\varepsilon}$, where $\tilde{k}=k\rho/c$. At steady state, the uninfected P cells are extinct as they are not renewed by any source. The U cells density is $U^{T}=\delta/\tilde{k}$, which is equal to both its pre-therapy level and its level under early-stage inhibition. The main difference between both scenarios is that the P cell expansion is replaced by an $I_{P}$-cell expansion, which induces a different viral set point. Similar to the early-stage case, the viral set point is independent of the proliferation function details and applies under general homeostasis conditions.

We are interested in comparing the viral loads that are attainable under both treatment types: $I_{_{U}}^{T}(1)=\frac{\lambda}{\delta(1-\varepsilon)}$ and $I_{_{U}}^{T}(2)=\frac{\lambda}{\delta}+\frac{\delta}{\tilde{k}}\times\frac{\varepsilon}{1-\varepsilon}$, where 1 and 2 denote the early-stage and late-stage approaches, respectively. It is easy to see that when $\varepsilon\to0$ we have $I_{_{U}}^{T}(1)\approx\frac{\lambda}{\delta}$, whereas $I_{_{U}}^{T}(2)=\frac{\lambda}{\delta}+\Delta$ with $\Delta=\frac{\delta}{\tilde{k}}\times\frac{\varepsilon}{1-\varepsilon}$. Since $\delta/\tilde{k}$ represents the U cell density in an infected individual, it is typically on the order of several hundreds of cells/μL, while $\lambda/\delta$ is approximately within the range of 10^-1^-1 cells/μL. That means that $\varepsilon$ must be very small in order for $\Delta$ to become comparable to $\lambda/\delta$. More precisely, we would like $\varepsilon$ to be sufficiently small such that $\frac{\lambda}{\delta}\approx\frac{\delta}{\tilde{k}}\times\frac{\varepsilon}{1-\varepsilon}\approx\frac{\delta}{\tilde{k}}\varepsilon$, which implies $\varepsilon\approx\frac{\lambda\tilde{k}}{\delta^{2}}$. For example, if we assign our default parameter values (see the Methods section in the main text) we obtain $\varepsilon\approx0.0013$. In general, $\varepsilon$ should be on the order of 10^-3^ or smaller if the bone-marrow’s relative contribution is on the order of a few percents. For larger$\varepsilon$’s, early-stage inhibition appears to be more advantageous in terms of viral suppression. Moreover, late-stage inhibition yields sustained improvements only for a narrow range of $\varepsilon$’s. Our bifurcation analysis indicated that for $\varepsilon$ as small as a few percents, this approach no longer results in a stable steady state with reduced viral loads and increased T cell count (data not shown). For example, in our default case, the bifurcation point occurs at $\varepsilon\approx0.0607$. Our analysis not only agrees with previous model-derived predictions [8], but also confirms that our much-simplified model captures the main finding of the more complex model in [8], and can be used for further investigation of gene therapy.

The weaker suppression that is achieved under late-stage inhibition reflects the limited expansion of infected P cells compared to the expansion of healthy P cells under early-stage inhibition. This, in turn, limits the pressure on the virus in the late-stage case. Importantly, the potency of late-stage inhibitors has not been quantified to date (*in vivo* or *in vitro*), and it is therefore not clear if they meet the above criterion or not. Estimating their potency in lab experiments is thus an important preliminary step in evaluating their applicability. Another concern with late-stage inhibition is the nature of the expanded cell population, since these cells already carry a suppressed provirus and may be re-infected. In such case, their protection might be weakened, a problem that is avoided with early-stage inhibition. Finally, recall that we analyzed an “ideal” case, whereby the virus is fully suppressed in infected P cells. Since the numbers of these cells rise dramatically due to their selective advantage, even residual viral production can make a difference in the overall effect on viral suppression (data not shown). Importantly, this is not the case under potent early-stage inhibition, where infected P cells are scarce. This further emphasizes the importance of assessing the inhibitory power of late-stage inhibitors on viral production and on the host cell functionality.

Our findings suggest that highly powerful inhibition of viral protein expression can exert therapeutic benefits that are comparable to those obtained when preventing viral entry. However, we stress that despite its disadvantageous viral suppression capability in the moderate potency regime, late-stage inhibition does result in considerable expansion of infected P cells (not shown). The resulting elevated T cell levels are crucial for HIV patients, as they postpone progression to AIDS. While our analysis focused on viral suppression, the latter effect should not be overlooked and may render late-stage inhibitors useful even in the moderate potency regime.

Dependence of the Suppression Gain on Thymus Contribution

We used Eq. (3) in the main text to illustrate the dependence of the viral suppression gain *G* on $\varepsilon$ and $\lambda$. Whereas changing $\varepsilon$ does not affect pre-therapy conditions, changing $\lambda$ results in a modified pre-therapy viral load ($I_{U}^{0}$) and a modified T cell level in a healthy individual, denoted by $T_{healthy}$. This is because both quantities are functions of $\lambda$. Since they also depend on the other blood-proliferation parameters, namely, *r* and *h*, one can compensate for the resulting changes by re-tuning *r* and *h* such that $I_{U}^{0}$ and $T_{healthy}$ remain fixed. Such re-tuning allows for a fair comparison between different relative thymus contributions. We adjusted the parameters by pre-setting the T cell level in the healthy state $T_{healthy}$, and subsequently setting$\lambda$ to equal the desired proportion of the natural T cell mortality per day, given by $dT_{healthy}$. Once$\lambda$ is calculated, it is left to solve for the values of *r* and *h* that satisfy the two steady-state equations describing $I_{U}^{0}$ and $T_{healthy}$. The stability of the post-therapy equilibrium and validity of calculated parameters were verified for each point shown in Figure 2B-2C in the main text.

Interestingly, the post-therapy viral load is independent of the proliferation parameters and function. Our results thus extend to any saturating rate function $g(U+P)$, provided that it results in a stable post-therapy set point. A natural candidate is logistic growth, which has been studied in the context of HIV models [9,10] and used in von Laer *et al.*’s model [8]. Simulations of our model with this function revealed similar dynamics, albeit much more oscillatory [11], and, as expected, similar viral load reductions. However, since increasingly larger suppression gains were accompanied by mounting oscillations, we found the Michaelis-Menten kinetics to provide a more biologically-relevant description.

**References**

1. Curlin ME, Iyer S, Mittler JE (2007) Optimal timing and duration of induction therapy for HIV-1 infection. PLoS Comput Biol 3: 1239-1256.

2. Venzke S, Michel N, Allespach I, Fackler OT, Keppler OT (2006) Expression of Nef Downregulates CXCR4, the Major Coreceptor of Human Immunodeficiency Virus, from the Surfaces of Target Cells and Thereby Enhances Resistance to Superinfection. J Virol 80: 11141-11152.

3. Chen J, Nikolaitchik O, Singh J, Wright A, Bencsics CE, et al. (2009) High efficiency of HIV-1 genomic RNA packaging and heterozygote formation revealed by single virion analysis. Proc Natl Acad Sci U S A 106: 13535-13540.

4. Wu H, Huang Y, Dykes C, Liu D, Ma J, et al. (2006) Modeling and estimation of replication fitness of human immunodeficiency virus type 1 in vitro experiments by using a growth competition assay. J Virol 80: 2380-2389.

5. Maree AFM, Keulen W, Boucher CAB, De Boer RJ (2000) Estimating relative fitness in viral competition experiments. J Virol 74: 11067-11072.

6. Perelson AS, Neumann AU, Markowitz M, Leonard JM, Ho DD (1996) HIV-1 dynamics in vivo: virion clearance rate, infected cell life-span, and viral generation time. Science 271: 1582-1586.

7. Speirs C, van Nimwegen E, Bolton D, Zavolan M, Duvall M, et al. (2005) Analysis of human immunodeficiency virus cytopathicity by using a new method for quantitating viral dynamics in cell culture. J Virol 79: 4025-4032.

8. von Laer D, Hasselmann S, Hasselmann K (2006) Impact of gene-modified T cells on HIV infection dynamics. J Theor Biol 238: 60-77.

9. Perelson AS, Nelson PW (1999) Mathematical Analysis of HIV-I: Dynamics in Vivo. SIAM Review 41: 3-44.

10. Perelson AS, Kirschner DE, De Boer R (1993) Dynamics of HIV infection of CD4+ T cells. Math Biosci 114: 81-125.

11. Wang L, Li MY (2006) Mathematical analysis of the global dynamics of a model for HIV infection of CD4+ T cells. Math Biosci 200: 44-57.
